# Decarbonizing the EU cement industry under technology and policy uncertainty

**DOI:** 10.1038/s41467-026-76611-3

**Published:** 2026-08-17

**Authors:** Paul Tautorat, Darius Sultani, Natalija Ljubic, Michael Pahle, Bjarne Steffen, Tobias S. Schmidt

**Affiliations:** 1 Energy and Technology Policy Group, Department of Humanities, Social and Political Sciences, ETH Zürich, Zürich, Switzerland; 2 Potsdam Institute for Climate Impact Research, Potsdam, Germany; 3 Department for Sustainable Energy Transition, Europa-Universität Flensburg, Flensburg, Germany; 4 Albert Einstein School of Public Policy, ETH Zürich, Zürich, Switzerland

**Keywords:** Climate-change mitigation, Climate-change policy, Energy modelling, Decision making, Climate-change mitigation

## Abstract

Cement production is a major source of CO_2_ emissions. In the European Union, the Emissions Trading System is the cornerstone instrument to incentivize industry decarbonization, including cement. However, major uncertainties prevail regarding allowance prices, carbon-capture investment costs, and the availability of CO_2_ pipelines and storage sites. These uncertainties affect real-world investment decisions, but are not yet well represented in models. Here, we develop a real options valuation model to assess how 179 cement plants across Europe respond to these uncertainties. Our results show that uncertainty perception strongly influences the timing and geography of investments. High confidence in favorable investment determinants could lead to widespread deployment of carbon capture and storage and biomass use, turning the cement industry into a carbon sink with 0.6 gigatons CO₂ removed between 2025 and 2050 in total. Low confidence, however, could lead many cement producers to delay action, resulting in cumulated emissions of 0.8 gigatons CO₂ over the same time frame. Biomass availability, financing conditions, and plant location further shape investment behavior, underscoring the need for credible energy and climate policy.

## Abstract (web summary)

The study models 179 European cement plants to see how uncertainty over carbon prices and technology costs affects low-carbon investment. Confidence in favorable conditions could make the industry CO_2_ negative by 2050; low confidence risks delays and higher emissions.

## Introduction

Cement is one of the most produced commodities worldwide. Due to its high carbon intensity, the industry contributes 8% of the global carbon emissions, making its fast and deep decarbonization imperative for reaching internationally agreed climate goals^1^. Cement production is particularly difficult to decarbonize due to its high temperatures and process emissions, making the use of carbon capture and storage (CCS) a likely option to achieve climate neutrality^1^. Combining CCS with a fuel switch to biogenic energy (BECCS) can permanently remove CO_2_ from the atmosphere. Therefore, cement production could become an important contributor to carbon dioxide removal (CDR), which would be in line with top-down models expecting BECCS to deliver most of the CDR by 2100^2^.

In the European Union (EU), the EU Emissions Trading System (ETS) sets the path to decarbonize the industrial sector by around 2040, when the final emission allowances will be issued^3^. Until then, the allowance cap will continue to gradually decline every year, making EU carbon pricing increasingly onerous for emitting installations. To help industry decarbonize, the EU is committed to supporting domestic investments through its recent Clean Industrial Deal, which foresees policies towards more affordable energy, demand side incentives, and over €100 billion in financing^4^. Nevertheless, key investment determinants for decarbonization remain uncertain^5^. They include future ETS allowance prices^6^, investment costs for low-carbon solutions^7^, and CO_2_ pipeline and storage site availability^8^. After years of incremental efficiency gains^1^, substantial decarbonization requires bulky, irreversible investments^9^, that may lead cement producers to delay investments amid uncertainty^10^.

While the major role of uncertainty in investment decisions is widely recognized^11^, insights into how uncertainty shapes temporal and geographical investment patterns related to decarbonizing the cement industry remain limited. Existing bottom-up investment models feature a detailed representation of technology, sector structure, costs, and policy^12–18^. They base investment decisions on criteria like payback times or total costs and incorporate uncertainty through (global) sensitivity analyses^19^. However, none of these models explicitly accounts for decision-making under (policy) uncertainty^20^. This gap leaves important questions unanswered.

First, given the landscape of cement plants today, it remains unclear to what extent the sector overall can contribute to mitigating climate change, both through emissions reductions and the potential to deliver CDR. To answer this question, the timing of investments is of particular interest. For the EU to meet its 2050 net-zero target, near-term investments are needed to comply with the steadily reducing ETS cap. Cement production is particularly affected by increasing ETS allowance prices caused by the cap reduction, due to cement’s high emissions intensity relative to its low general cost^21^. However, a lack of credibility of EU climate policy^6^ may lead cement producers to delay investments. A lack of investments could lead to the perpetuation of emissions, increased allowance prices, and eventually political opposition to the ETS system. This political opposition could ultimately lead to the EU weakening its climate policy ambition^22^.

Second, more clarity is needed on the distributional implications of a “new economic geography of decarbonization”^23^—the spatial redistribution of low-carbon production capacities across regions—in the European cement industry. For this, it is relevant to consider which regions in Europe are well-positioned to host future low-carbon cement plants, benefiting from favorable biomass and CO_2_ transport conditions, and which plants may struggle to transition in time. Today, cement production is more geographically dispersed than other carbon-intensive industry sectors, such as steel or chemicals, due to regionally distributed demand and high transport costs relative to the value of the product^21^. Beyond transport costs, there is substantial variation in the locational factors across European cement plants. Against this background, there is limited understanding of how accounting for decision-making under uncertainty influences the geographical aspects of the cement industry’s decarbonization^24,25^.

Third, the choice of decarbonization option—particularly whether including a fuel switch to biomass or not—affects both potential deliveries of net-negative emissions and the extent to which biomass demand from low-carbon cement would put pressure on agriculture and further ecosystem services. While competition for biomass and the risk of excessive biomass use have been widely discussed in the literature^2^, dedicated analyses on potential biomass demands from the cement industry are still lacking.

We address these questions by modeling the investment decisions of the European cement industry at the plant level, considering how uncertainties in the investment landscape around the decarbonization options CCS, biomass (BIO), and their combination (BECCS) influence low-carbon investment. We do so by developing a pure jump real options valuation (ROV) model^20,26,27^ to assess the decarbonization investment behavior of 179 integrated cement plants across Europe, more specifically across all countries that are regulated under the EU and the Swiss ETS. In the model, plant characteristics are varied across four dimensions: production capacity^28^, CO_2_ transport and storage costs – which we refer to as transport costs from here on^29^, financing costs^30^, and local biomass costs (“Methods”)^31^. Our plant-level focus allows us to evaluate geographical patterns of low-carbon cement investment, but requires us to abstract from additional abatement options in the construction value chain such as Supplementary Cementitious Materials or demand-side reduction^13^. In addition, we exclude kiln electrification from the analysis because of its inability to address process emissions, as well as its relative technological immaturity^32^. The chosen real-options approach enables us to estimate the value of waiting with an irreversible investment, and to identify the effect of key uncertainties on timelines for decarbonization investments in the EU cement industry, including variations in producers’ expectations about the future development of key investment determinants.

With ETS allowance prices being a key driver of investment decisions, 17% of cement production is expected not to be decarbonized by 2050, amid concerns about a potential weakening of EU climate policy stringency. However, we also show that the ETS has the potential to fully decarbonize the cement industry early on and deliver substantial negative emissions. For this to happen, policymakers would need to reinforce the credibility of the CO_2_ price signal and associated regulations, while also considering the impact on biomass demand.

## Results

### Uncertain investment determinants

**Fig. 1:**
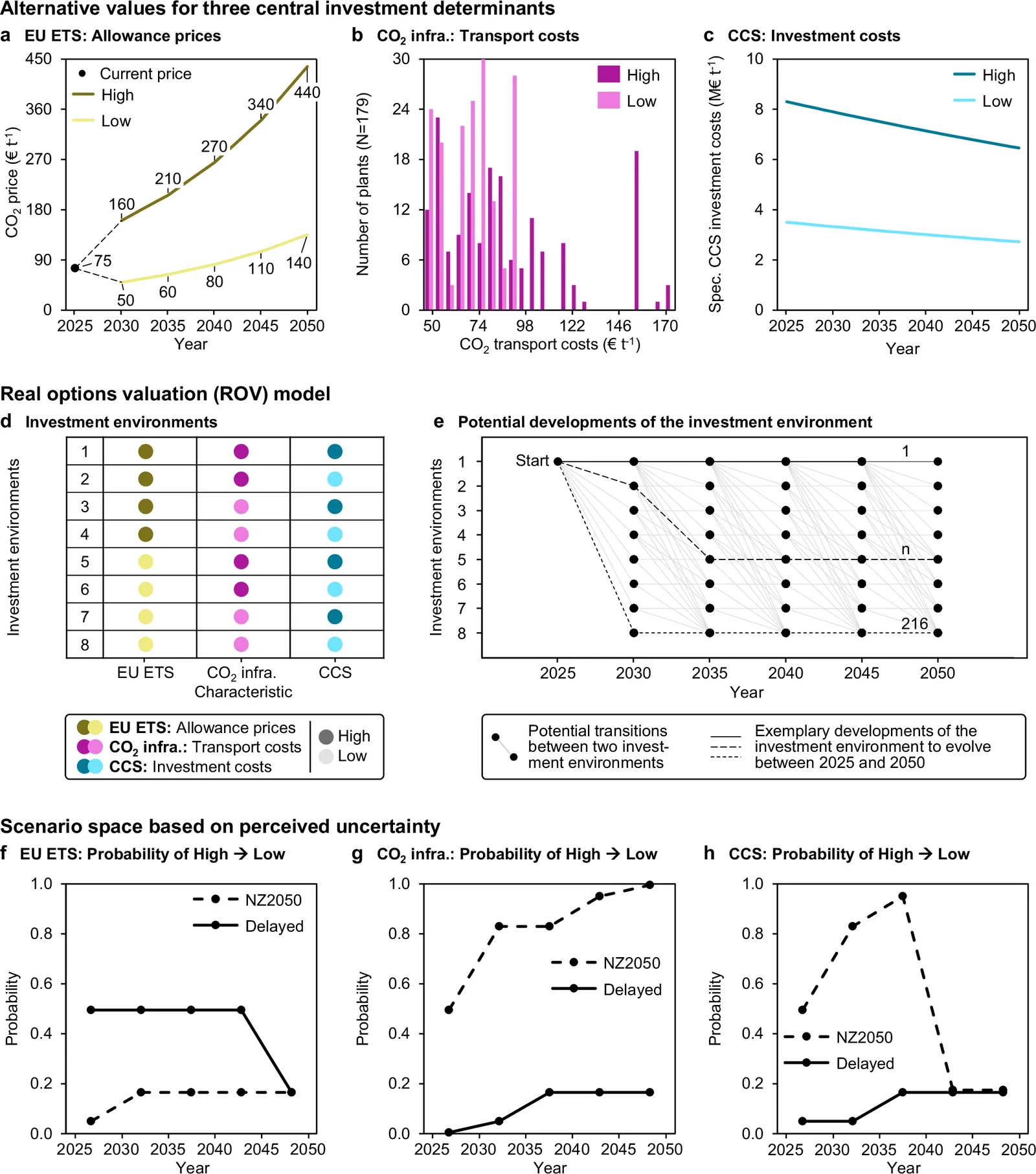
Uncertain investment determinants and respective probabilities form the ROV model’s investment environment. **a** Plants face the same alternative values for ETS allowance prices. Depending on the investment environment, this is either an ambitious (High) or less ambitious (Low) price, as per LIMES-EU modeling results^3^. **b** CO_2_ transport costs vary across plants depending on the availability of a CO_2_ transport infrastructure (pipelines and storage sites), with the buildout of infrastructure reducing the costs from High to Low. **c** The investment costs of carbon capture and storage (CCS), indicated as the specific investment costs in M€ per ton of CO_2_ captured per hour, can reduce from the High to the Low value in line with technology projections from the industry^33^. **d** Investment decisions are modeled across eight investment environments, which are characterized by the EU ETS allowance prices, CO_2_ transport costs, and CCS investment costs. **e** Investment decisions are modeled between 2025 and 2050 using a pure jump ROV model, which allows for the investment environment to change every five years along a lattice through a combination of three events: First, a reduction of the ambition level of the ETS; second, the buildout of CO_2_ transport infrastructure in Europe; and third, the reduction of CCS investment costs. At the start in 2025, the ETS is ambitious, there is no CO_2_ transport infrastructure, and CCS investment costs are high. **f–h** ETS participants and cement producers have different perceptions of the probability of the investment environment changing. The probability ratings from the interviews are differentiated into the two scenarios, NZ2050 and Delayed. The NZ2050 scenario combines low probability ratings for a less ambitious ETS with high ratings for an infrastructure buildout and reduced investment costs. The Delayed scenario combines high probability ratings for a less ambitious ETS with low ratings for infrastructure buildout and investment cost reductions.

We conceptualize uncertainty for Europe’s cement industry through (i) alternative values for three key investment determinants and (ii) cement producers’ probability ratings for these alternative values to materialize: ETS allowance prices^3^, location-specific CO_2_ transport costs^29^, and specific CCS investment costs^33^. For each of these determinants, we introduce a set of (high) reference and another set of (low) alternative price/cost values as depicted in the upper row of (Fig. 1a–c, and “Methods”), covering a wide but realistic range. The combination of these alternative values for the three determinants results in eight (2^3^) different investment environments (Fig. 1d). As these can change over time, and as we cover 25 years in five-year time steps, our model considers 216 potential developments of the investment environment (Fig. 1e and “Methods”). These are probability-weighted to incorporate cement producers’ perception regarding the probability that the determinants change between the alternative values in each time step. In expert interviews, ETS participants and stakeholders from the European cement industry rated the respective probabilities of a change in each of the three investment determinants over time (“Methods” and Supplementary Methods). For the three uncertainties represented, we hence elicit firms’ expectations of the future investment environment from interviews with industry practitioners. Figure 1f–h shows the results of these interviews, where probability ratings are differentiated into two scenarios. The Net Zero 2050 (NZ2050) scenario represents the probability that the respective determinant has a favorable value for decarbonization at each point in time (e.g., high ETS allowance prices); the Delayed scenario represents the probability that the respective determinant has an unfavorable value for decarbonization (e.g., low ETS allowance prices).

### Investment patterns and emissions trajectories

**Fig. 2:**
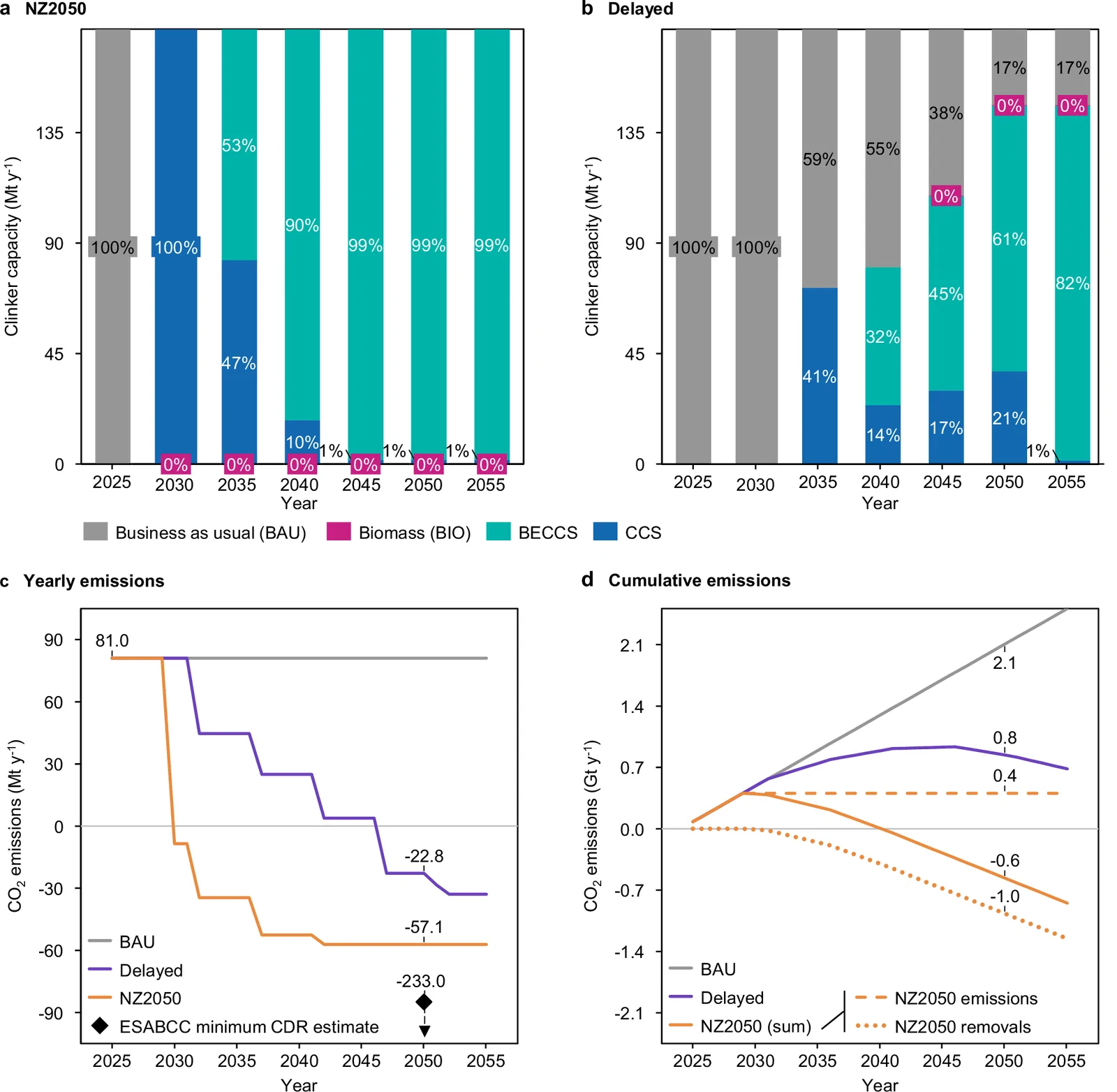
Temporal deployment patterns by technology and respective emissions trajectories. Temporal development of technology stocks in the European cement industry for NZ2050 (**a**) and delayed (**b**) scenario. The indicated clinker production capacities are the sum of probability-weighted nameplate capacities for all 216 conceivable developments of the investment environment. Displayed 0%-labels are rounded. Yearly (**c**) and cumulative (**d**) emissions from the European cement industry for the NZ2050 and Delayed scenarios compared to emissions following business as usual (BAU). For 2050 in (**c**), the minimum estimate for required Carbon Dioxide Removal (CDR), including bioenergy with carbon capture and storage (BECCS) and direct air carbon capture and storage (DACCS), from the European Scientific Advisory Board on Climate Change (ESABCC) is indicated, exceeding net-negative emissions in the NZ2050 and Delayed scenarios by factors of 4 and 10, respectively^34^. In (**d**), emissions for NZ2050 are further broken down into emissions from 2025 to 2030 and CDR from 2030 to 2050, allowing for a better comparison with literature estimates of CDR. The indicated CO_2_ emissions are the sum of probability-weighted emissions for all 216 conceivable developments of the investment environment. Additionally, emissions were normalized using the average of allowances surrendered by the cement plants in the sample between 2020 and 2023, to account for plant utilization.

To illustrate the range of possible investment patterns, we present two alternative scenarios. The NZ2050 scenario assumes that the EU will adhere to its 2050 net-zero target, reflecting investors’ high probability ratings of favorable investment determinants, i.e., high ETS allowance prices, the availability of CO_2_ infrastructure (i.e., pipelines and storage sites), and reductions in CCS investment cost. In contrast, the Delayed scenario represents high probability ratings of lower ETS allowance prices, delayed or no CO_2_ infrastructure, and no progress in CCS investment costs. In sum, these scenarios highlight the divergent perceptions of uncertainty among industry practitioners, which lead to different investment behavior, emissions pathways, and consequently the sector’s long-term role as an emissions source or sink. More specifically, low-carbon investment patterns of European cement plants are predominantly driven by producers’ confidence in high ETS allowance prices, as well as in the (future) development of CO_2_ transport infrastructure (Fig. 2a, b).

In the NZ2050 scenario, where confidence in EU climate policy is high, producers swiftly opt for CCS, and then gradually combine it with a fuel switch to biomass to generate negative BECCS emissions thereafter. In this more optimistic scenario, the model fully retrofits the sector to CCS by 2030 already. The fact that we do not nearly observe such investment activity in the real-world could—aside from supply-side limitations—reflect producers’ lack of confidence in a steep and sustained rise in ETS allowance prices, as well as in the timely deployment of the required CO_2_ infrastructure.

In the Delayed scenario, where confidence in EU climate policy is lower, producers amounting to 17% of overall clinker capacity choose not to invest at all, opting instead to continue purchasing ETS allowances even in 2050. Producers with favorable conditions regarding financing, biomass, and CO_2_ transport transition to CCS as early as 2035. The remaining producers delay CCS/BECCS investments until later in the transition, waiting for higher ETS allowance prices and less uncertainty.

The scale and timing of low-carbon investments have a critical influence on the cement industry’s emissions and CDR until 2050, and therefore impact whether its cumulative emissions over the next three decades are positive or negative^34,35^. In our NZ2050 scenario, the cement industry transitions to a sink in the early 2030 s already and contributes 57.1 megatons (Mt) of annual CDR by 2050 (Fig. 2c). This means the cement industry alone could deliver half of the EU’s annual industrial CDR in 2050 as projected by the EU Commission’s 2040 target Impact Assessment^36^. In sum, it would then generate net-negative emissions of 0.6 gigatons (Gt) by 2050, with 1 Gt being CDR (Fig. 2d). If EU climate policy is weakened or lacks confidence (Delayed scenario), on the other hand, the European cement industry would cumulatively emit 0.8 Gt of CO_2_ by 2050 (Fig. 2d). This is despite its transition from an emissions source to a sink of 22.8 Mt of CO_2_ annually in 2050 (Fig. 2c), which comes too late to compensate previous emissions during the period from 2025 to 2045.

We lack installation-specific data on plant age, but conduct a stylized sensitivity analysis in Supplementary Note 1. The NZ2050 scenario results are robust both for older and newer plant fleets, indicating that high confidence in the investment environment provides plant operators with a compelling business case regardless of plant age. In the Delayed scenario, however, an initially older plant fleet both accelerates the transition slightly and lands at a lower overall emission level; a newer plant fleet shows the opposite effect.

### Investment by country and plant

**Fig. 3:**
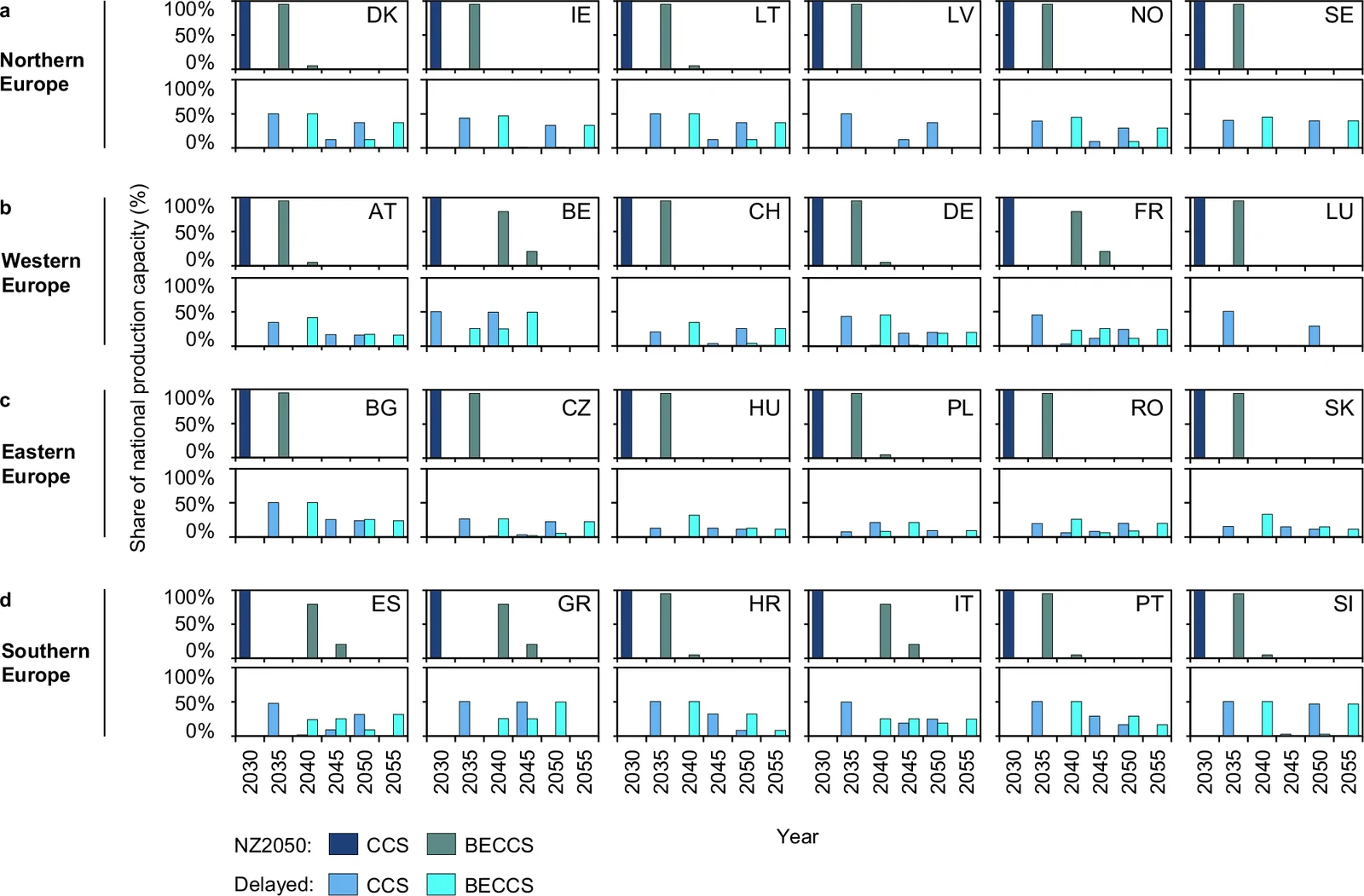
Temporal deployment patterns per country and technology. Temporal deployment patterns by country for the NZ2050 (upper half per panel) and Delayed (lower half) scenarios are presented as the share of national production capacity. The modeled cement plants are located across 24 countries (the ISO-2 country code is indicated top right per panel), which are clustered into four country groups: Northern (**a**), Western (**b**), Eastern (**c**), and Southern Europe (**d**). The values for bioenergy with carbon capture and storage (BECCS) either indicate a joint investment in carbon capture and storage (CCS) and biomass, or an investment in biomass following a previous CCS investment.

From a geographical perspective, our results highlight the risk of smaller and landlocked plants being left behind due to a combination of modeled uncertainties and adverse locational factors in a world with ambitious carbon management. In other words, the observed investment probabilities allow us to derive regions with favorable conditions for a low-carbon cement industry in Europe. While in our model CCS is deployed in all plants by 2030 under the NZ2050 scenario (upper half of each panel in Fig. 3), the timing of biomass integration depends on (country-specific) biomass costs. As a result, countries in Northern Europe experience an early transition, while countries like Spain, Italy, and France undergo a later transition.

In the Delayed scenario, countries with close carbon sinks and low biomass costs gain a stronger competitive edge over the rest of Europe, and the variation between countries becomes more pronounced (Fig. 3). Among the 83% of producers that invest in CCS or BECCS (Fig. 2b), those that switch early in 2035 and with a high probability are typically located in countries with both low CO_2_ transport costs, even without dedicated pipelines and additional storage sites, and competitive biomass costs.

**Fig. 4:**
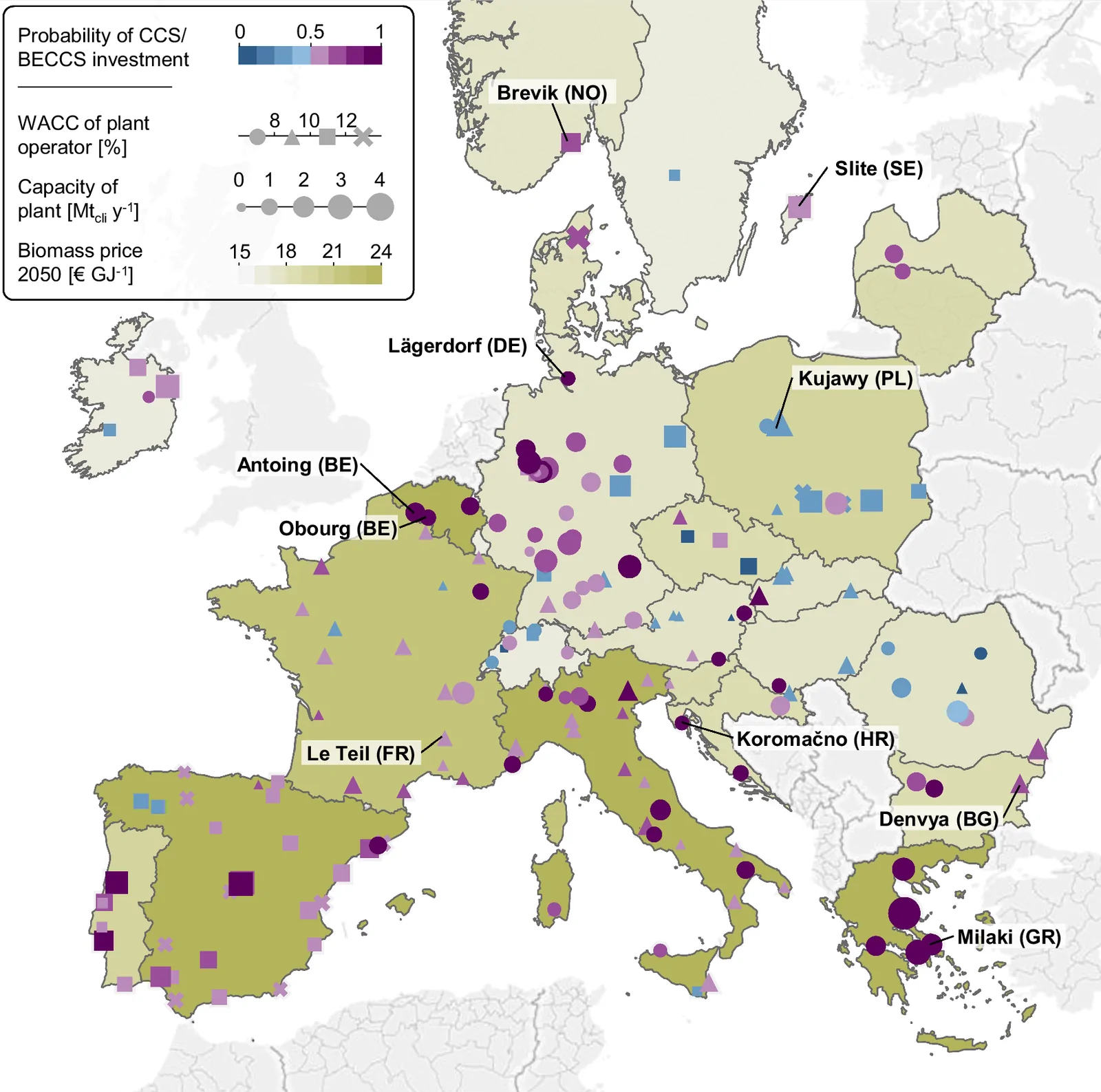
Investment patterns for the delayed scenario in 2040 at the plant level. Each icon represents one cement plant. The color of each icon indicates the probability of the producer investing in carbon capture and storage (CCS) or bioenergy with carbon capture and storage (BECCS) by 2040. The shape of each icon indicates the producer’s weighted average cost of capital (WACC). The size of the icons indicates the plant’s nameplate capacity. The different shades of green indicate the 2050 biomass price per country. Map data copyrighted to OpenStreetMap contributors^60^. The Netherlands and Estonia are grayed out because there is no integrated cement plant in these countries. The United Kingdom is grayed out as its emissions trading system is not linked to the EU ETS yet. Labels are assigned to those plants for which Heidelberg Materials and Holcim, major cement producers, announced CCS installations by 2030. Heidelberg Materials aims to achieve net zero by 2050 and plans to install most of its European CCS facilities before 2030 at coastal plants in Slite (SE), Brevik (NO), and Devnya (BG), or near waterways, such as in Antoing (BE), which aligns with our model’s plant-level results^37^. As a first step, the company has installed a pilot CCS unit at its Brevik plant, targeting a 50% reduction in emissions^37^. These sites all benefit from low CO_2_ transport and biomass costs. Similarly, Holcim plans to install its first six CCS units by 2030, at plants in Koromačno (HR), Milaki (GR), Le Teil (FR), Obourg (BE), Lägerdorf (DE), and Kujawy (PL). For the Kujawy plant only, the model projects an investment probability below 50%, likely because it is the only plant not located near the sea or major waterways. However, Kujawy is the largest cement plant in Poland, making it an interesting location because of scale-effects.

Additionally, we observe considerable within-country variance of investment decisions in the Delayed scenario. This is mainly driven by locational factors, which play a relatively larger role in the investment decision vis-à-vis low ETS allowance prices. At the plant level, notable clusters with higher (purple in Fig. 4) and lower probabilities (blue in Fig. 4) emerge. For example, producers in Greece are projected to invest with the highest probability. These plants benefit from large production capacities (0.7–3.4 Mt of clinker per year), which generate scale effects for CCS investment costs. Additionally, they face low financing costs (6–7%), and their coastal location enables low-cost CO_2_ transport by ship. The availability of offshore storage at the Prinos oil field in the nearby Aegean Sea^29^ further supports investment in these plants. In contrast, plants in landlocked regions in countries such as Switzerland, Austria, Poland, Hungary, and Romania are less likely to invest due to their dependence on uncertain ground-based CO_2_ transport infrastructure. Smaller plants with higher investment and financing costs also have lower investment probabilities. Our results align well with the current decarbonization plans announced by major European cement producers like Heidelberg Materials and Holcim^37^.

### Biomass demand for cement decarbonization

**Fig. 5:**
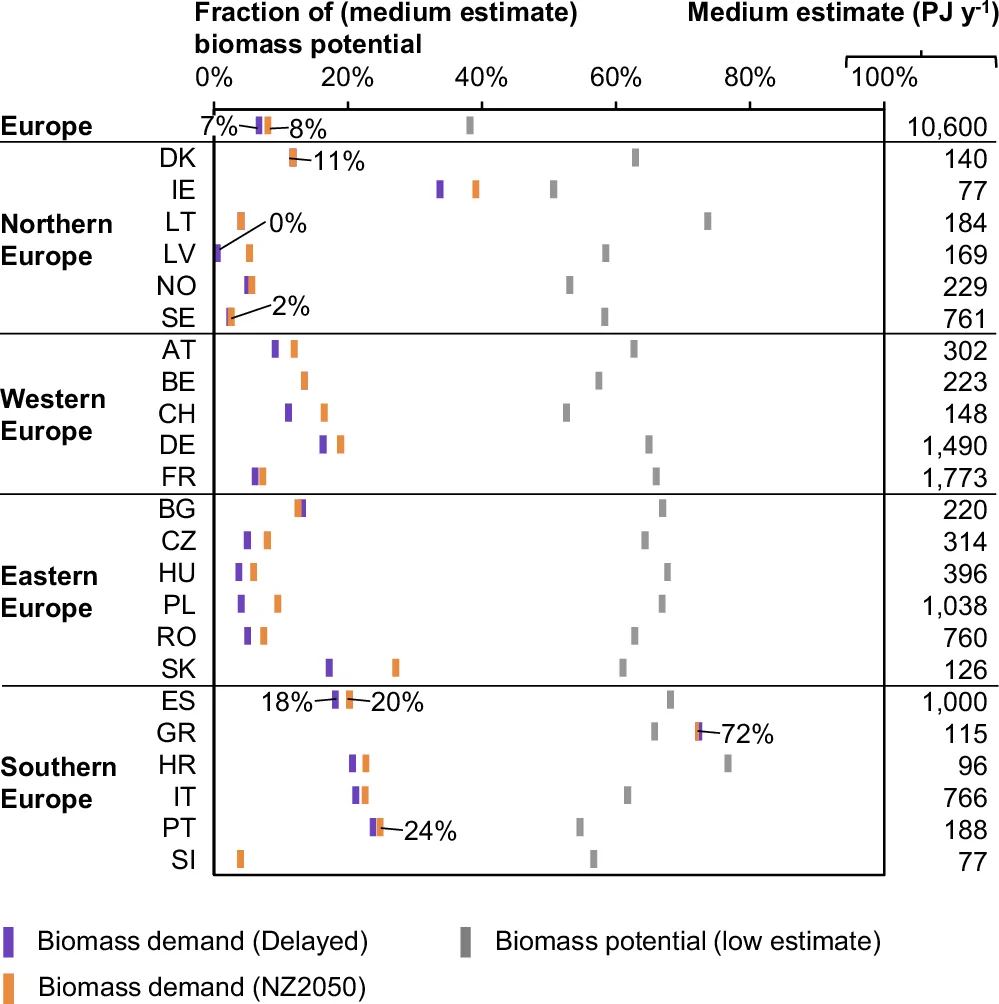
Biomass demand from cement production vs. country-level biomass potentials. Comparison of the biomass demand of the European cement industry in 2050 (for bioenergy with carbon capture and storage (BECCS) deployment in the Delayed and NZ2050 scenarios) with national biomass potentials (low and medium estimates from the ENSPRESO database^31^). The modeled cement plants are located across 24 countries, which are clustered into four country groups: Northern, Western, Eastern, and Southern Europe. Per country, the absolute values for the medium estimate of the biomass potential are indicated on the right (equivalent to 100%). The top line, “Europe,” indicates demand and potential summed for all countries and normalized using the average of allowances surrendered by the cement plants in the sample between 2020 and 2023 to correct for plant utilization. Values for Luxembourg were removed due to the country’s limited biomass potential of 8 PJ y^−1^.

Cement decarbonization may use a substantial portion of Europe’s locally available biomass, depending on the country. BECCS could enable a net-zero and net-negative future for the cement industry and unlock the carbon (removal) value of biomass, which is considered higher than its energetic value from a societal perspective^38^. BECCS deployment under the EU ETS, such as in the case of cement, would come with the additional advantage that the compliance market mechanism would allocate biomass to the activity that achieves a unit of mitigation, both abatement and removal, at the lowest cost. However, switching large parts of cement production capacity to BECCS will put pressure on European biomass supply, warranting a comparison of future biomass demand and potential. Assuming a current biomass use from cement of approximately 90 PJ per year across Europe, we project a potential eight- to nine-fold increase until 2050^13,39,40^. In our model, the switch to BECCS results in the cement industry demanding a substantial share (8% in sum) of the European local biomass potential (NZ2050 for the top line in Fig. 5). However, there are major differences between countries: In the Northern European countries Denmark (DK), Lithuania (LT), Latvia (LV), Norway (NO), and Sweden (SE), switching all cement plants to BECCS (as the case for NZ2050) would demand only 2% (SE) to 11% (DK) of their estimated national biomass potentials. However, in Southern European countries, demand would increase to 20% of the national potential of Spain (ES) and 24% of Portugal (PT), respectively. For Greece (GR), this share would rise to 72%, even exceeding the low estimate for the biomass potential in the ENSPRESO database^31^.

For the Delayed scenario, the overall demand from the cement industry would be smaller but still considerable (7% in sum). Here, the variation between countries is even larger, with some fully transitioning to BECCS and others only deploying CCS without biomass use (Latvia; 0% of potential). Deploying less mature oxyfuel CCS or direct capture instead of the modeled amine scrubbing technology, which cement producers are also considering, would enable a shift from biomass to electricity, reducing pressure on biomass supply^41^. While the cement industry will inevitably compete for this biomass with other sectors, cement producers have the advantage of being able to handle different types of (low-quality) biomass^33^.

## Discussion

Our results highlight that confidence in favorable investment determinants is essential to develop the European cement industry into a carbon sink quickly. We find that the industry should favor locations with low CCS and biomass costs; however, actual distributional outcomes depend on producers’ confidence in the transition. Additionally, biomass demand from cement plants could reach critical levels in the EU, and there are large differences between Member States’ abilities to meet this demand with sufficient local supply. The removal volumes observed in the NZ2050 scenario amount to roughly a quarter of annual CDR (BECCS + DACCS), which the European Scientific Advisory Board on Climate Change considers necessary to reach the 2 °C target^34^. However, for widespread BECCS deployment to occur, several conditions must be met.

First, policies need to be put in place to incentivize first waves of CCS deployment in the cement sector now. Since EU ETS prices can only provide sufficient incentives in the mid to long term, ambitious Member States should facilitate early abatement through the provision of instruments like Carbon Contracts for Difference (CCfDs)^42,43^, as well as a coherent long-term strategy for CO_2_ infrastructure. Such policies could be complemented by regulatory requirements to install CCS on new cement plants, as well as additional measures to increase biomass supply. To provide a long-term perspective for investors, the EU also needs to uphold and strengthen the credibility of its carbon market^44^, particularly by maintaining an ambitious ETS allowance price in the face of political resistance^45^. To safeguard the integrity of the ETS, the EU should reach consensus on a clear target picture for the time after an “ETS endgame.”^46^ One recently proposed alternative foresees the international linking of industry-specific trading systems, which could provide a more targeted approach for specific sectors such as cement^47^. Additionally, the EU needs to uphold a functioning regime against carbon leakage^48,49^, more specifically, to support the ETS credibly through the implementation of the Carbon Border Adjustment Mechanism (CBAM)^50,51^.

Second, clearer political guidance is needed on the role of cement in delivering negative emissions, as a complete transformation would place considerable pressure on European biomass markets. Even under the Delayed scenario, substantial BECCS deployment and biomass demand remain plausible (7% of the medium estimate). This demand would inevitably lead to rivalry with other sectors (e.g., heating or sustainable aviation fuels). The simplest economic solution to handle tradeoffs between CDR, agriculture, and ecosystem services would be to price biomass externalities appropriately, ensuring it is used where it has the highest societal value. While such a policy could change the picture we have drawn, further analysis is required, which is beyond this work’s scope. An initial analytical step could be developing a “biomass ladder,” following the example of the well-established hydrogen ladder^52^, which could serve as a starting point for prioritization discussions and eventually lead to a European biomass strategy.

Third, it is essential to consider the geographical aspects of decarbonization. Under the Clean Industrial Deal and Just Transition Mechanism, the EU has two options for supporting the cement industry. A biggest-bang-for-the-buck approach would focus support on large plants near carbon sinks and affordable biomass, which already have competitive advantages. Future work can contribute to more accurately identifying such competitive plants by increasing installation-specific data transparency, specifically on OPEX and plant age. Yet these sites would likely be prioritized by international multi-plant producers anyway, and such concentration risks geographical restructuring, potentially undermining cohesion and provoking backlash^53^. Alternatively, supporting also higher-cost locations, such as through de-risking grants or concessional loans, could help preserve the industry structure and reduce risks to the ETS and the broader policy framework. The EU could also fund pipelines and storage sites in regions with high CO_2_ transport costs^8^. We do not propose a whatever-it-takes approach, as due to cement production overcapacities, some consolidation remains unavoidable, as recent plant closure announcements indicate^54^.

Beyond these policy implications, our ROV model showcases more generally how uncertainty and investors’ confidence in climate policy influence bulky, irreversible investments, as applicable in a wide range of other industries and countries^55,56^. However, cement, being easier to decarbonize than other industries, such as aviation or chemicals^57^, is expected to decarbonize relatively early, serving as a litmus test for the credibility of EU climate policy.

## Methods

This study first compiles plant-level data of all 179 integrated cement plants regulated under the EU and Swiss emissions trading systems. The plants differ with regard to production capacity^28^, CO_2_ transport and storage costs^29^, financing costs^30^, and country-specific biomass costs^31^. Second, we develop a real options valuation (ROV) model that accounts for investment flexibility in the face of uncertain policy, technology, and infrastructure developments to assess low-carbon investment dynamics in the European cement industry under uncertainty. Third, we perform two rounds of expert interviews: the first to validate our investment assumptions and to identify relevant technological and policy uncertainties, and the second to quantify perceived uncertainties and inform scenario probabilities in the model. The Methods section is structured along these three steps.

### Plant and techno-economic data

#### Cement plants in Europe

We compiled a plant-level dataset of cement production installations regulated under the EU and Swiss emissions trading systems. Starting with the “List of operators in the EU” from the Union Registry (June 2022), we filtered installations by activity type (“Production of cement clinker”) and status (“ACTIVE”), resulting in a list of ETS-regulated clinker producers. While some individual cement plants report under different activity codes and are therefore not part of the data set (e.g., the Finnsementti plant in Lappeenranta, Finland), this approach provides us with a systematic and reproducible selection algorithm that avoids contaminating the sample with non-cement producers. This list was complemented with emissions data from the EU ETS transaction log (April 2025), yielding 232 active installations with associated information on installation names, registry member state, ETS ID, and historical CO₂ emissions. We then integrated six additional Swiss cement plants, which are covered by the EU ETS-linked Swiss ETS, based on publicly available information. After merging both datasets, we applied a final round of filtering to exclude and correct installations that had closed, did not produce ordinary Portland cement (OPC), or operated multiple kiln lines under a single ETS ID. The final sample includes 179 integrated cement plants.

#### Plant locations, CO_2_ transport costs, and production capacities

Based on available data regarding plant characteristics, we distinguish between cement facilities across four dimensions: CO2 transport costs, production capacities, cost of capital, and country-specific biomass cost. All other techno-economic parameters, both in terms of cost and technology-specific operating parameters, are assumed to be homogenous across all plants. To capture site-specific cost heterogeneity, we manually matched ETS installation names with facility names in a dataset provided by the non-governmental organization CATF in May 2023^29^, which includes georeferenced data on CO₂ transport and storage costs across Europe. This allowed us to assign location coordinates and CATF transport cost estimates to each plant. Using plant locations, we then matched each facility with the geoasset database for cement from the Spatial Finance Initiative (SFI)^28^ to retrieve nameplate production capacity. Reported capacities were cross-validated against past emissions to flag implausible values, which were manually checked and corrected via desk research. Future changes in production capacities, driven by shifts in cement demand and current overcapacity, would add another layer of complexity to investment decisions, but are not explicitly modeled. Final capacity estimates were used to adjust investment cost assumptions based on economies of scale, and technology learning was assumed in line with literature values to reflect incremental innovation over time (Fig. 1c)^58,59^. More detailed information on our data is provided in Supplementary Methods 2.

#### Cost of capital

Financing conditions were derived using a multi-step approach, resulting in a plant-level distribution of the weighted average cost of capital (WACC) ranging from 4% to 14%. First, we searched for Bloomberg tickers associated with each plant’s installation name. If a ticker was found, we extracted WACC data for the period 2013–2022 using Bloomberg’s BDH function and calculated the mean WACC over the period. For plants lacking a direct ticker, we identified the global ultimate owner (GUO) via the Orbis database and used Bloomberg data for the parent company. In the 85 cases where no plant- or firm-specific WACC was available, a fallback estimate based on country averages—or the sample average—was applied. These values were further validated through desk research.

#### Biomass cost per country

Biomass costs were determined on a national level to reflect current and future biomass costs. We used long-term biomass price projections from the REMIND integrated assessment model developed at the Potsdam Institute for Climate Impact Research (PIK), which are used as input to the LIMES-EU model. To introduce geographical differentiation, we combined these with country-level biomass cost estimates from the ENSPRESO database, which aggregates costs across 17 biomass feedstocks at the national level^31^. We refer the reader to Supplementary Figs. 4 and 5, as well as Supplementary Table 5 for additional information on our biomass assumptions.

#### Other techno-economic data

Investment and operational parameters for amine-based CCS were sourced from the European Cement Research Academy. The default ETS allowance price values (“high ETS allowance price values”) and the alternative (“low ETS allowance price values”) are based on the LIMES-EU model, assuming different linear reduction factors for the ETS cap^3^. Technical specifications for kiln fuel mixes, potential for biomass substitution, and thermal efficiency were assumed to be technology-specific but homogenous across plants and based on academic and gray literature^33^. CO₂ transport and storage costs are plant-specific and reflect estimates from the CATF data^29^, under the “short-term infrastructure” scenario in which only limited onshore and offshore storage capacity is assumed to be available. The alternative case assumes dedicated pipelines have been built and additional storage sites have been developed, significantly reducing transport and storage costs.

### Real options valuation (ROV) model

#### Model overview

We developed an ROV model to assess the timing and probability of low-carbon investment decisions by individual plant operators under uncertainty, which is largely based on the model introduced by Tautorat et al.^20^. The model is separately applied to cement plants across 24 European countries, each defined by plant-specific techno-economic parameters, including nameplate production capacity, CO₂ transport and storage costs, biomass costs, and the WACC. Unlike traditional discounted cash flow approaches, which select technologies based on static net present value (NPV) comparisons, our model captures the value of flexibility in delaying or adjusting investments in response to evolving policy and technological conditions. For each plant, the producer can choose between investing in CCS, biomass combustion (BIO), their combination (BECCS), or continuing with the incumbent fossil-based setup. Decisions are taken at five-year intervals from 2025 to 2050, with investment cash flows evaluated through 2079 to reflect technology lifetimes.

#### Uncertainty structure

To reflect structural uncertainty and informed by our expert interviews, we model three sources of uncertainty: (1) the ambition level of EU climate policy (affecting ETS allowance prices), (2) the availability of CO₂ transport infrastructure (affecting transport costs), and (3) deployment of and innovation around CCS technologies (affecting CCS investment costs reductions). To reflect producers’ perceptions of uncertainty, each of these factors has a specific probability of changing from a reference value (R) to an alternative value (A) at each time step, but not back. At each point in time, a producer’s investment environment is thus characterized by a specific combination of values for the three investment determinants, resulting in eight (2³) possible combinations. Starting in 2025, the ETS allowance prices are high, CO₂ transport costs are high, and CCS investment costs are high. The investment environment can change through changes from these reference values to the alternative values for each of the three uncertain investment determinants. Assuming these events are irreversible, five junctures and three events lead to 216 (6^3^) possible combinations (or potential developments of the investment environment) between 2025 and 2050, each with varying probability ratings.

#### Cash flow and emissions

Cash flows are modeled relative to a fossil-based baseline and include five components: investment costs (CAPEX), operation and maintenance (O&M) costs, fuel costs (FU), and (where applicable) CO₂ transport and storage costs (ST). Emissions reductions contribute to the investment case by sparing the producer from having to surrender Allowances at the current ETS allowance price, ultimately reducing emissions costs (EM). An additional green premium—i.e., a higher willingness to pay for low-carbon cement downstream the value chain—is not considered. Negative emissions from BECCS generate an additional revenue stream from removal credits, which are also valued at the respective ETS allowance price at each time step. While this could be achieved by making carbon dioxide removal (CDR) eligible within the ETS^35^, other policy instruments that value a permanent removal accordingly would lead to the same financial outcome.

#### Calculation of net present values

The expected NPV of a decision is calculated by summing discounted future cash flows over all future periods and possible state transitions as Eq. (1) (Supplementary Methods 3). For example, in the last time step, no further uncertainty exists, and the NPV simplifies to the sum of discounted deterministic cash flows. For earlier periods, future states are weighted using transition probabilities surveyed through interviews. Discounting of cash flows is based on the plant-specific WACC as described above.

1 $${NPV}=\Delta {EM}-\Delta \left({CAPEX}+O{{\&}}M+\,{FU}+{ST}\right)+{option}\,{values}$$

#### Option values

The model incorporates two types of real options: the option to delay (or wait) and the option to follow up with additional investments. Option values are calculated recursively and discounted using plant-specific WACCs to the respective decision point using future state transition probabilities.

#### Payback and NPV criteria

Investment options are only considered if they satisfy a maximum payback period, reflecting investor preferences for near- to mid-term returns. Specifically, only opportunities with a positive cumulative NPV over the first 15 years (including construction time) are eligible, which is slightly higher than the numbers reported for less transformative investments, e.g., into energy efficiency. Additionally, from the set of eligible investment options at each decision point, the plant selects the one with the highest total NPV, which includes both the expected cash flows and any option values for future follow-on actions.

### Interviews with industry experts

#### Technologies and investment determinants in the cement industry

To inform our model design and parameterization, we conducted a first round of semi-structured interviews with 13 experts from across the European cement industry between 2021 and 2024. Interviewees were selected based on their expertise in investment planning, technology deployment, and participation in the carbon market. Participants were contacted with a short project summary and invited to participate voluntarily. Those who agreed received an interview guide in advance. Interviews were conducted online or in person by members of the research team (PT, DS, NL, BS, TS), and each interview’s duration ranged from 16 to 120 minutes, with a median of 45 to 60 minutes. The Chatham House Rule was followed to ensure open discussion. The interviews focused on identifying the most salient investment uncertainties from an industry perspective—such as carbon pricing, infrastructure availability, and capital costs—as well as validating and refining the techno-economic assumptions used in the ROV model.

#### Alternative ETS allowance price values and probability ratings

A second round of semi-structured interviews was conducted with 14 experts in 2023 and 2024 to quantify their probability ratings for key events modeled in the ROV framework. Specifically, interviewees were asked to assess the probability of three developments: (1) a reduction in EU ETS allowance prices due to weakening policy ambition, (2) the availability of dedicated CO₂ transport infrastructure, and (3) a decline in capital costs of CCS technologies. Alternative ETS allowance price values under a less ambitious climate policy scenario were also surveyed (Supplementary Table 4). However, due to a large variance in surveyed ETS allowance price values, we instead base the alternative (lower) ETS allowance price values in our modeling on the same source (i.e., the LIMES-EU model) as the reference values^3^. The alternative values are based on a scenario that resembles the ETS before the 2023 reform and is more consistent with price levels observed at the time of writing. These ETS price values are substantially lower, allowing for a more comprehensive price range. Participants were contacted in advance with a project summary, and the interviews were structured along an interview guide. Interviews were conducted by members of the research team (PT, DS, BS, TS) and lasted between 30 and 60 minutes, either online or in person. The Chatham House Rule was followed to ensure open discussion. Interviewees were encouraged to comment only on those uncertainties where they felt confident.

## Supplementary information

Supplementary information

Transparent Peer Review file

## Data Availability

The data used for Fig. 1a–c of this study are publicly available from Pahle et al.^3^ (a), CATF^29^ (b), and ECRA^33^ (c), respectively. The data used for Fig. 1f–h are assumptions informed through expert interviews and are included in the Supplementary Information. Map data for Fig. 4 copyrighted OpenStreetMap contributors and are available from https://www.openstreetmap.org/copyright.^60^ The data generated in this study for Figs. 2–5 have been deposited in GitHub at https://github.com/paultautorat/cementROV^61^.

## Code availability

The code to reproduce the analysis is available via GitHub at https://github.com/paultautorat/cementROV^61^.
