# Supplementary material for: Decarbonizing the EU cement industry under technology and policy uncertainty
Source: Nat Commun. 2026 Aug 17;17:9862. doi: 10.1038/s41467-026-76611-3 (PMC13578682; doi:10.1038/s41467-026-76611-3)
Supplement: Supplementary file 1 [file 41467_2026_76611_MOESM1_ESM.pdf]

Supplementary Information

Decarbonizing the EU cement industry under technology and policy uncertainty

Paul Tautorat[1], Darius Sultani[2,3], Natalija Ljubic[1], Michael Pahle[2],
Bjarne Steffen[1,4], Tobias S. Schmidt[1,4,*]

[1] *Energy and Technology Policy Group, Department of Humanities, Social and Political Sciences, ETH Zürich, Switzerland*; [2] *Potsdam Institute for Climate Impact Research, Potsdam, Germany*; [3] *Department for Sustainable Energy Transition, Europa-Universität Flensburg, Germany*; [4] *Albert Einstein School of Public Policy, ETH Zürich, Switzerland*; *Corresponding author: tobiasschmidt@ethz.ch

Supplementary Items

Supplementary Methods **3**

Supplementary Notes **24**

Supplementary Methods

Supplementary Methods 1: Interviews with industry experts

Round 1 Technologies & Uncertainties: Interviewees

To inform our model design and parameterization, we conducted a first round of semi-structured interviews with 13 experts from across the European cement industry between 2021 and 2024 (cf. Supplementary Table 1 for a list of interviewees).

Supplementary Table 1: List of interviewees from round 1

No.	Type of organization	Role of interviewee
1	Research group 1	Researcher
2	Research group 2	Researcher
3	Plant operator 2	Senior Researcher
4	Industry association 1	Head of Communication and Public Affairs
5	Industry association 2	Director/Managing Partner/CEO
6	Non-governmental organization/Think-tank	Senior Technical Manager
7	Cement producer 1	Reearch/Technology Manager
8	Cement producer 1	Director Competence Center
9	Cement producer 2	(Deputy) Plant Manager
10	Cement producer 2	Research/Technology Manager
11	Cement producer 3	Research/Technology Managerr
12	Cement producer 4	(Deputy) Plant Manager
13	Cement producer 5	Director Sustainable Development

Round 1 Technologies & Uncertainties: Questions

The interviews focused on identifying the most salient investment uncertainties (referred to as *investment determinants* in the Main) from an industry perspective—such as carbon pricing, infrastructure availability, and investment costs—as well as validating and refining the techno-economic assumptions used in the real options valuation (ROV) model.

1. Are the model and its results in this form relevant for cement industry practitioners?
2. Is our choice of decarbonization options (focus on FS [fuel switch] and CCS) sensible?
3. How relevant is the EU ETS price as a decision criterion?
4. What other factors do you consider when choosing between decarbonization options (economic and non-economic)?
5. Is our "reference cement plant" a good approximation to use in a model for the European cement industry?
6. Which options [technologies] for carbon capture do you see as the most promising for the cement industry?
7. When do you expect them [carbon capture technologies] to become commercially available?
8. Would you consider our cost assumptions for carbon capture and alternative fuel options realistic?
9. Are 5 and 2 years realistic assumptions for construction times for CCS and a fuel switch, respectively?
10. Do you have any questions or comments on the interview?
11. Can you recommend experts in the cement industry that we should talk to?

Round 2 ETS allowance prices & probability ratings: Interviewees

A second round of semi-structured interviews was conducted with 14 experts in 2023 and 2024 to quantify probability ratings for key events modeled in the ROV framework (cf. Supplementary Table 2 for a list of interviewees).

Supplementary Table 2: List of interviewees from round 2

No.	Type of organization	Role of interviewee
1	EU ETS participant (non-cement) 1	Head of Energy and Climate Policies
2	EU ETS participant (non-cement) 1	Head of Carbon Markets
3	Non-governmental organization/Think-tank 1	Policy Director
4	Non-governmental organization/Think-tank 1	Expert on EU Industrial Decarbonization
5	Non-governmental organization/Think-tank 2	Project Manager Industry
6	Non-governmental organization/Think-tank 3	Director/Managing Partner/CEO
7	Non-governmental organization/Think-tank 4	Director/Managing Partner/CEO
8	Cement producer 1	Global Lead CCUS
9	Cement producer 5	Head of Innovation
10	Cement producer 5	Research/Technology Manager
11	Cement producer 5	Research/Technology Manager
12	Cement producer 6	Sustainable Development
13	Cement producer 7	CEO
14	Cement producer 7	CFO

Round 2 ETS allowance prices & probability ratings: Questions

The interviews focused on an alternative ETS allowance price curve (cf. question 1 below) and assessing probability ratings for three developments (cf. questions 2–4 below): (1) a reduction in EU ETS allowance prices due to weakening policy ambition, (2) the availability of dedicated CO_2 transport infrastructure, and (3) a decline in investment costs of CCS technologies. More specifically, the following questions were asked (exemplary for the first period from 2025 to 2029):

1. Assume EU climate action remained ambitious until 2025 (cf. solid line). In case of EU climate policy being loosened (e.g., through the reduction of the linear reduction factor (LRF)), to which level do you expect the ETS price to drop, i.e., **what is an alternative "politically feasible" ETS path?**

2. Assume EU climate action remained ambitious until now (cf. solid line). What is the probability of EU climate action to slow down between 2025 and 2029? The slow down would result in a lower EUA price. A previous elicitation of experts familiar with the ETS and the political process around it, resulted in the depicted alternative pathway (cf. dotted line).**What is the probability of EU climate action to slow down between 2025 and 2029?**

3. Assume the price of transporting and storing CO_2 for cement and steel plants in Europe is on average 81 EUR/tCO_2 as no dedicated CO_2 pipelines are available yet. What is the probability of CO_2 pipelines becoming available in Europe between 2025 and 2029? CO_2 pipelines would significantly reduce the costs of transporting CO_2 leading to costs of only 65 EUR/tCO_2 in 2030 and 55 EUR/tCO_2 in 2035 compared to 69 EUR/tCO_2 without infrastructure.**What is the probability of CO_2 backbone becoming available in Europe between 2025 and 2029?**

4. Assume the specific CAPEX of a post combustion CCS plant are on the level of ECRA's high estimate, i.e., 275 MEUR/(6,000tcli/year). What is the probability of the CAPEX to turn out to be rather on the level of ECRA's low estimate, i.e., costs of 200 MEUR/(6,000tcli/year)?**What is the probability of CAPEX to turn out much lower between 2025 and 2029?**

Round 2 ETS allowance prices & probability ratings: Replies

Jump probabilities as given in Supplementary Table 3 are assumed based on expert interviews (cf. Methods and Fig. 1 f–h in the Main). α indicates the probability of a less ambitious ETS; β indicates the probability of an infrastructure buildout; and γ indicates the probability of reduced investments costs.

Supplementary Table 3: Surveyed probability ratings

Period t	Years	Probability ratings					
		NZ2050			Delayed		
		α	β	γ	α	β	γ
1	2025-2029	0.05	0.50	0.50	0.50	0.01	0.05
2	2030-2034	0.17	0.83	0.83	0.50	0.05	0.05
3	2035-2039	0.17	0.83	0.95	0.50	0.17	0.17
4	2040-2044	0.17	0.95	0.18	0.50	0.17	0.17
5	2045-2050	0.17	1.00	0.18	0.17	0.17	0.17

Alternative ETS allowance price values under a less ambitious climate policy scenario were also surveyed until 2050 (cf. question 1 above and Supplementary Table 4). However, due to a large variance in surveyed ETS allowance price values, we instead base the alternative (lower) ETS allowance price values in our modeling, just like the reference values, on a scenario from the LIMES-EU model[1] that resembles the ETS before the 2023 reform and is more consistent with price levels observed at the time of writing. These ETS price values are substantially lower, allowing for a more comprehensive price range.

Supplementary Table 4: Reference and alternative EU ETS allowance price values in EUR_{2023}/tCO_2

Year	LIMES-EU		Expert interviews
	Reference values	Alternative values	Alternative values
2030	160	50	100
2035	206	64	123
2040	265	82	146
2045	340	105	173
2050	436	135	204
2055	560	173	
2060	574	626	
2065	601	626	
2070	664	637	

Supplementary Methods 2: Data

Plant-level costs

Plant characteristics are varied across four dimensions: production capacity, CO_2 transport (and storage) costs, financing costs, and local biomass costs. This subsection covers the first three dimensions, biomass is described in the following subsection. All remaining OPEX components are assumed to be symmetrical across firms, and are based on the European Cement Research Academy (ECRA)[2]. Based on a Cemsuisse report[3], we furthermore assume an initial fuel mix comprised of fossil waste (51.5 %), coal (30 %), biomass (18 %), and some minor additions of natural gas and fuel oil (<1 %, respectively).

To get a first overview of the data spread, Supplementary Figure 1 shows the distribution of plants across financing costs and CO_2 transport costs.

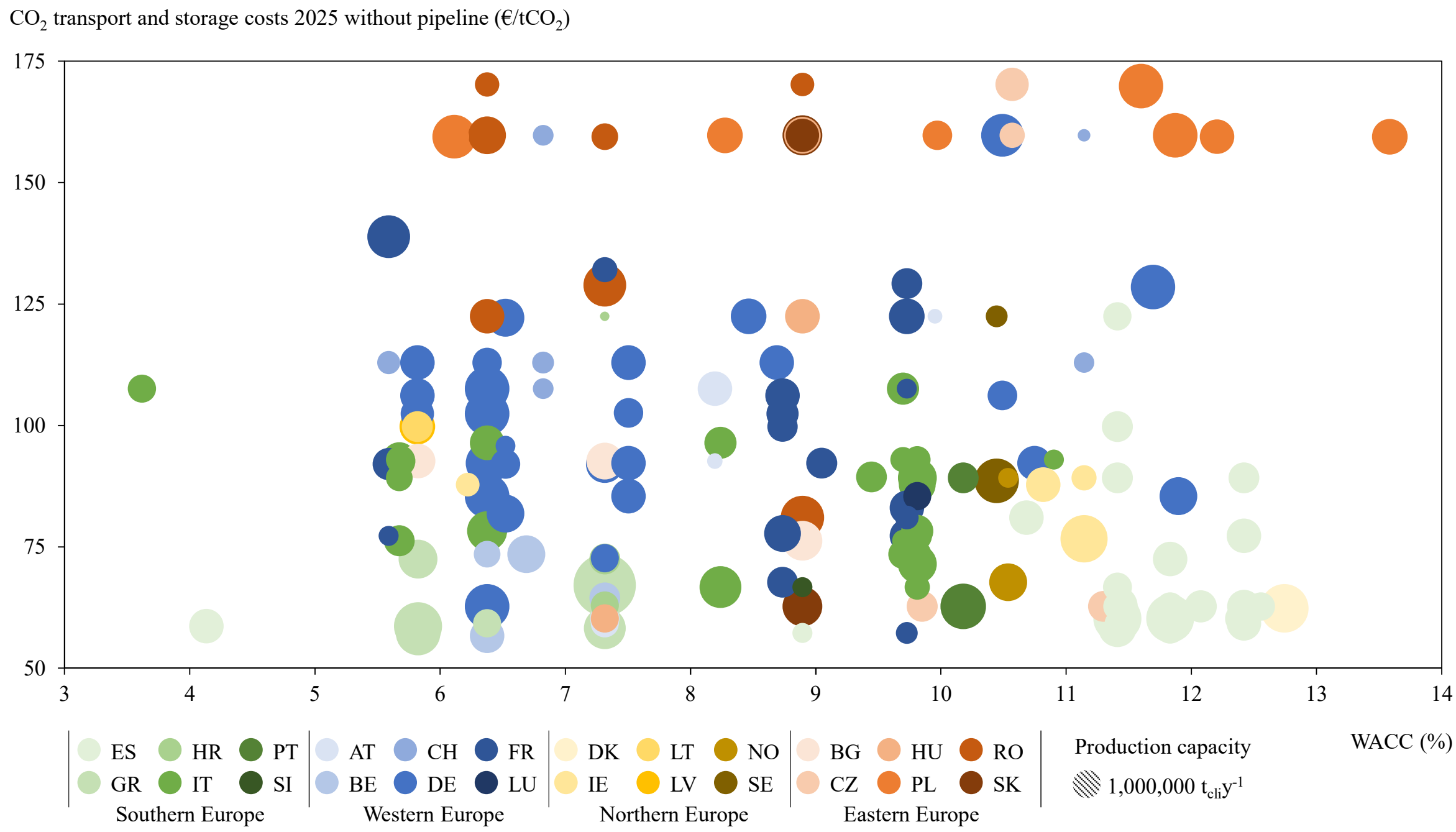

Supplementary Figure 1: **Spread of cement plant data points.** Scatterplot of plant data points with respect to financing cost (represented as the Weighted Average Cost of Capital) and costs for CO_2 transport in 2025. The bubble size represents yearly production capacity, which we harness to reflect economies of scale in our capital expenditure assumptions (see below).

The uncertainties on CO_2 transport cost and CAPEX introduced in our model lead to a wide variety of potential cost paths over time. For simplicity, for these two parameters we compare the starting point across all plants, i.e. consider the cost input for 2025 that – chronologically speaking – firms base their initial investment decisions on. In correspondence with the vertical axis of Supplementary Figure 1, Supplementary Figure 2 displays the spread of CO_2 transport cost across all 179 plants in our data set.

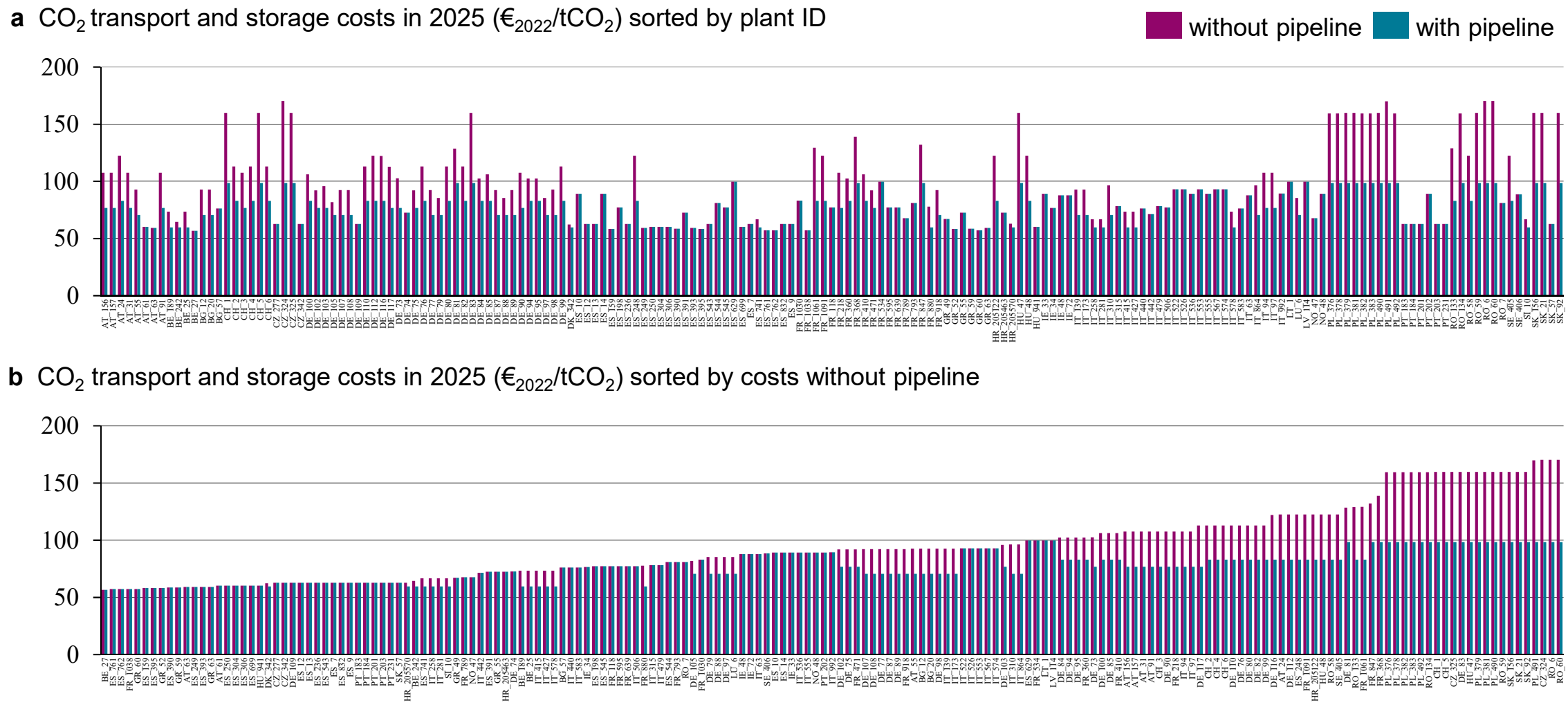

Supplementary Figure 2: **Distribution of CO_2 transport costs.** CO_2 transport costs – sorted by plant ID (a) and costs without pipeline (b) – across all our 179 plants in the set in 2025, based on Clean Air Task Force (CATF).[4]

Moving on to CAPEX for CCS, we use a plant's yearly production capacity (bubble sizes in Supplementary Figure 1) to reflect economies of scale in the investment. As shown in Supplementary Equation (1), for each plant p we take a basic scaling approach with scaling factor $\theta = 0.6$ to scale a reference plant $\bar{p}$ from ECRA[2] with capacity $\bar{Q}$ and CAPEX $\bar{I}$ to the respective size of p.

$$I_p = \bar{I} \cdot \left(\frac{Q_p}{\bar{Q}} \right)^{\theta} \tag{1}$$

The resulting CAPEX distribution for 2025 across all plants in the set is visualized in Supplementary Figure 3. We reflect the possibility of disruptive innovation through the CAPEX uncertainty inherent to the model, i.e. with the surveyed jump probabilities to the low-CAPEX path. In addition, incremental innovation is modelled as a yearly CAPEX decrease of 1% over the entire time frame (Figure 1c).

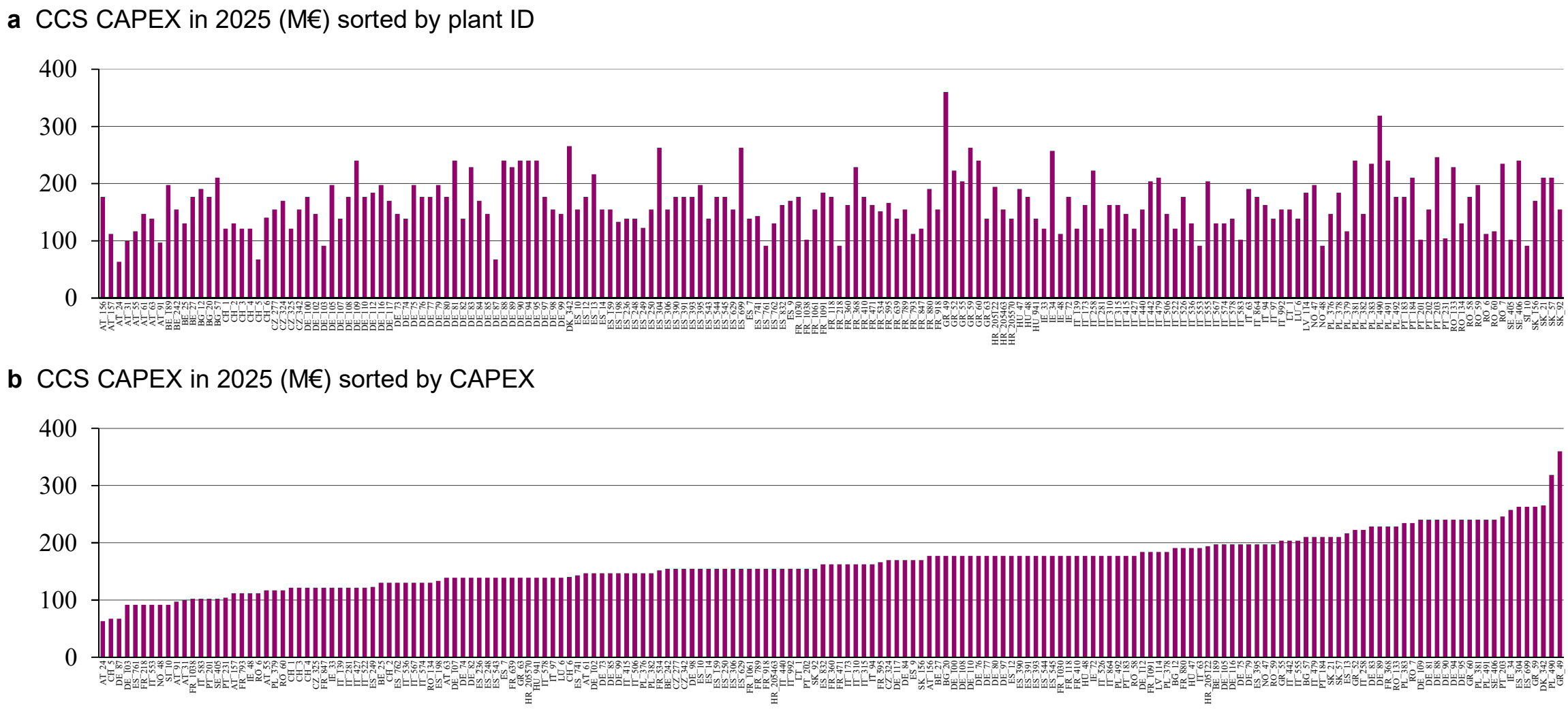

Supplementary Figure 3: **Capital expenditure (CAPEX) distribution.** Capacity-scaled CAPEX in million Euros – sorted by plant ID (a) and CAPEX (b) – across all our 179 plants in the set in 2025, based on ECRA.[2]

Biomass potentials and costs

We include 17 biomass types as reported in the ENSPRESO dataset.[5] The respective potentials per country are displayed in Supplementary Figure 4.

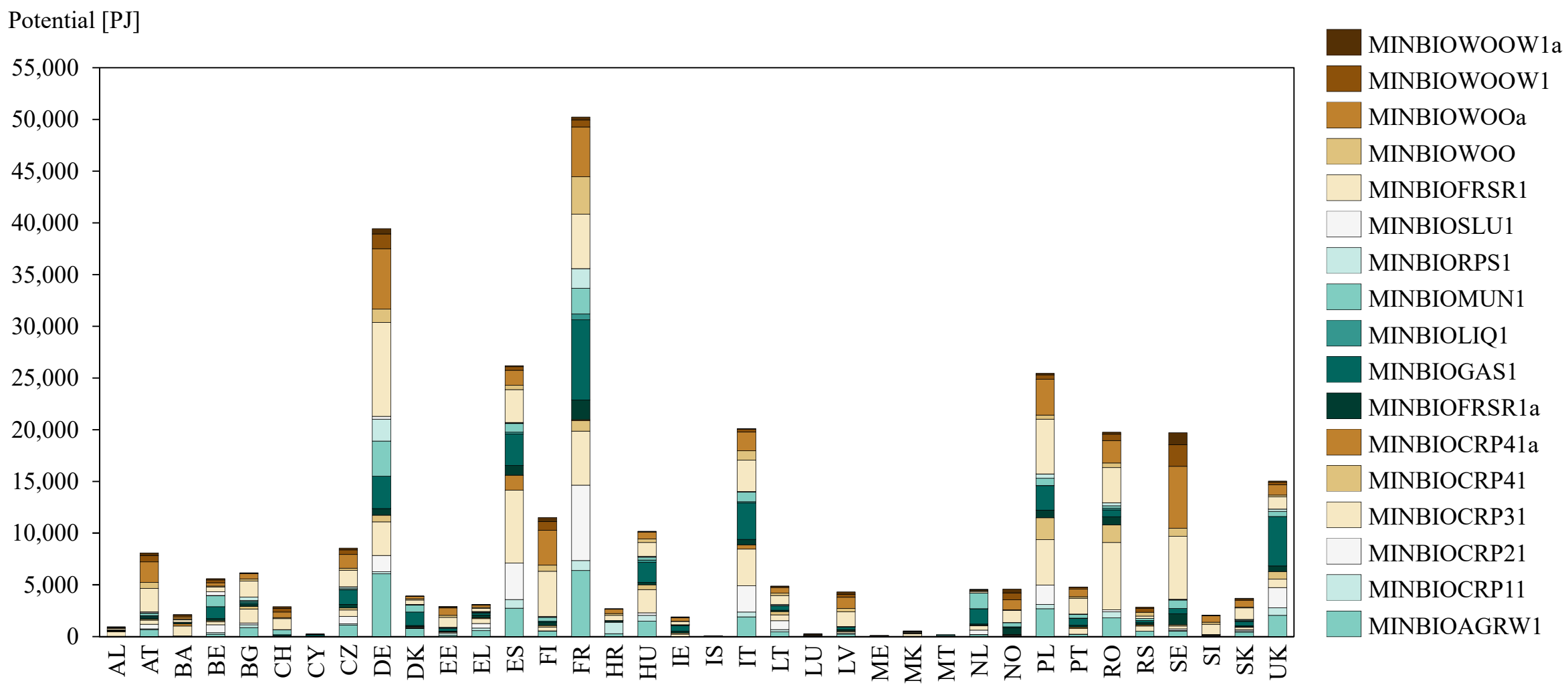

Supplementary Figure 4: **National biomass potentials.** ENSPRESO biomass potentials per country across 17 biomass types.

Code explanations per biomass type are given by Supplementary Table 5.

We combine two sources of biomass cost assumptions to derive our cost input for the model. First, the ENSPRESO dataset reports cost per different biomass types, which allows us to assume a cost spread across different biomass inputs.[5] However, ENSPRESO does only report supply curves and

Supplementary Table 5: ENSPRESO biomass type codes as displayed in Supplementary Figure 4

code	biomass type	group
MINBIOWOOW1a	sawdust	forestry
MINBIOWOOW1	woodchips	forestry
MINBIOWOOa	chips and pellets	forestry
MINBIOWOO	fuelwood	forestry
MINBIOFRSR1	fuelwood residues	forestry
MINBIOSLU1	sludge	-
MINBIORPS1	rape seed	energy crops
MINBIOMUN1	municipal waste	-
MINBIOLIQ1	sunflower, soya seed	energy crops
MINBIOGAS1	manure solid, liquid	agriculture and landscape residues
MINBIOFRSR1a	residues from landscape care	agriculture and landscape residues
MINBIOCRP41a	poplar	energy crops
MINBIOCRP41	willow	energy crops
MINBIOCRP31	miscanthus, switchgrass, reed canary grass	energy crops
MINBIOCRP21	sugar from sugar beet	energy crops
MINBIOCRP11	barley, wheat, grain maize, oats, other cereals and rye	energy crops
MINBIOAGRW1	agricultural waste	agriculture and landscape residues

does not take demand-side competition for biomass between various sectors into account.

To close this gap, we utilize biomass price levels from REMIND as a median estimate, around which we then apply the ENSPRESO cost spread. This way, we take demand for biomass from all sectors into account, assuming price formation that is closer to an actual supply-demand equilibrium. The resulting values are reported in Supplementary Figure 5.

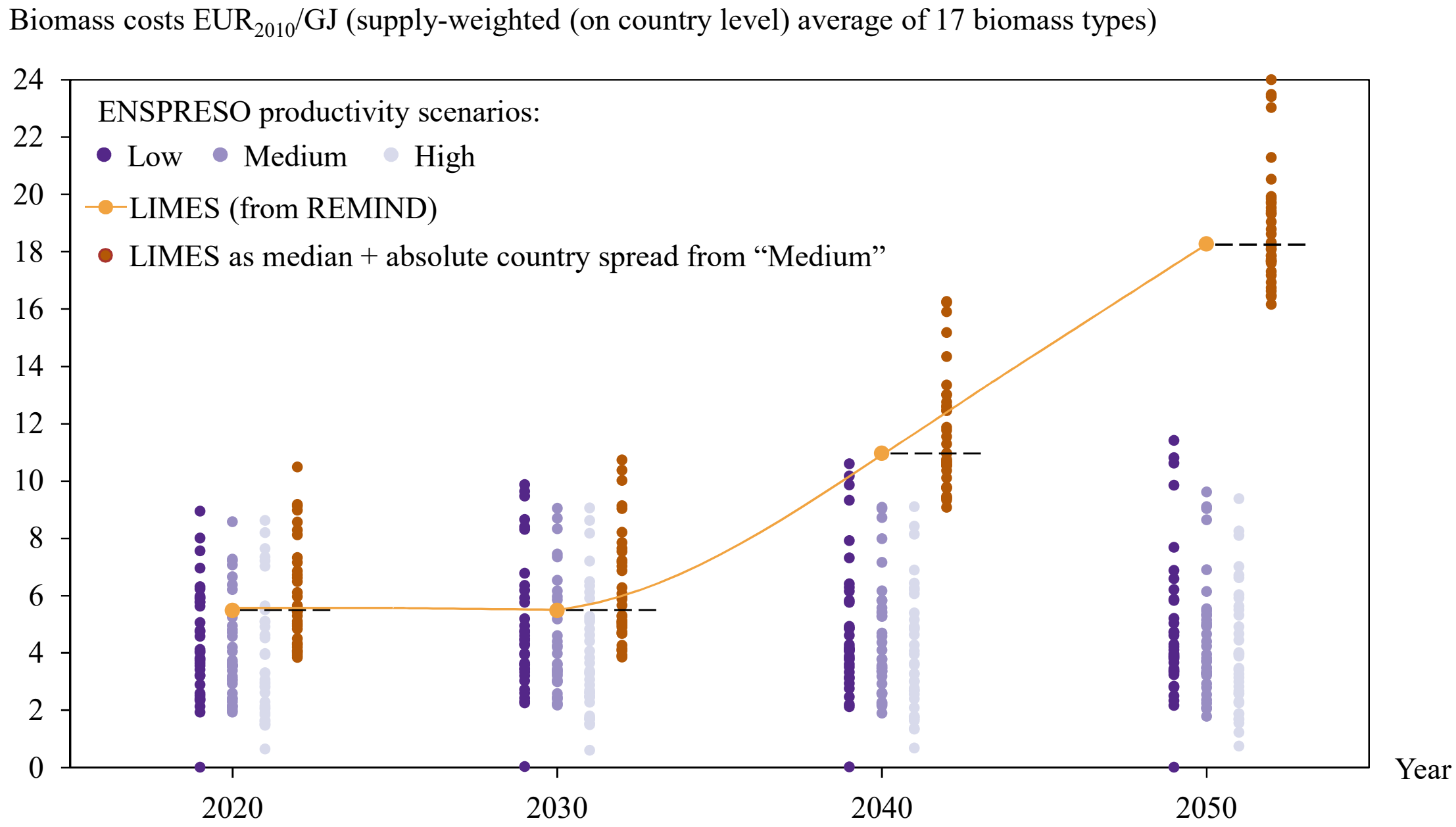

Supplementary Figure 5: **Biomass cost.** Biomass cost assumptions as a combination of the REMIND price level and the ENSPRESO cost spread per country.

Supplementary Methods 3: Model description

We develop a ROV model to illustrate the decarbonization investment behavior of all 179 integrated cement plants across Europe. Since we aim to systematically assess the impact of uncertainties, we expand on conventional discounted cash flow approaches by taking option values of potential investments into account. To do so, we introduce three uncertainties in the investment environment: (i) ETS prices, (ii) CO_2 pipeline and storage availability (reflected in CO_2 transport cost), and (iii) CAPEX. For each of these three uncertainties, we derive a reference path from the literature as a starting point. In a nutshell, this starting point (i.e. the investment environment today) is characterized by ambitious ETS prices, but higher cost for CO_2 transport and storage, as well as higher CAPEX. Uncertainty is then represented as follows: As time progresses, future states of these three characteristics could result in an irreversible jump from the reference path to an alternative price/cost path. From our interviews with practitioners, we construct two scenarios – NZ2050 (i.e. more optimistic) and Delayed (i.e. rather pessimistic) – for how respective jump probabilities develop over time. Given these uncertainties, the model allows us to quantify the option values of waiting and add-on investments, which are taken into account on top of the net present value to arrive at the investment decision.

The following detailed model description is an adaption of the description published by Tautorat et al. (2025).[6] In each state (referred to as *investment environment* in the Introduction and Results sections) $sc \in SC = \{1, ..., 8\}$ and period $t \in T = \{1, ..., 6\}$ of length $L_1 = 5a$ each plant $p \in P = \{1, ..., 6\}$ takes the irreversible decision $d \in D_p = \{1, ..., 10\}$ that maximizes its expected profits over all modeled years $y \in Y = \{1, ..., 55\}$ by either reducing its emissions (costs) through one or a combination of decarbonization technologies or by waiting and emitting as before until the next period. Plants are assumed to take decisions in the first 25 years between 2025 and 2050. To account for differences in technology lifetimes, an additional 30-year period until 2079 is modeled, leading to 55 years in total. Each plant's decisions are assumed to be independent of other plants. For an investment opportunity to be chosen, it has to meet a specific payback time c_p (cf. *Payback criterion*) and have the lowest costs of all opportunities (cf. *NPV criterion*).

The model takes into account the value of the option to wait and the option of follow-on investments (cf. *Option values*), which go back to the model's ROV-logic that, starting from a *reference* state in the first period, the state can jump (referred to as *change* in the Main) between every two subsequent periods (cf. *States, uncertainties, and jump probabilities*) and thereby make (follow-up) investments potentially more favorable in the future. The model is implemented as a lattice model (cf. Fig. 1e) that is solved recursively to account for option values of investment opportunities in the future. More specifically, jumps between states are modeled to influence the cash flows of the incumbent setup (i.e., existing rotary kilns with a defined fuel mix) and those resulting from the decarbonization technologies.

Calculation of NPVs

The net present value $\overline{NPV}^{sc}_{t,p,d}$ of investment opportunity d taken by plant p at the beginning of period t and in state sc is the sum of discounted future cash flows CFs until the end of the modeling horizon, which are weighted for all future states as specified in Supplementary Equation (2) and visualized in Supplementary Figure 6. $\overline{NPV}^{sc}_{t,p,d}$ structurally differs for different periods: For $t = |T|$ (=6), the ultimate period to invest in, it is the sum of discounted certain future CFs until the end of the modeling horizon as no further jumps between states are assumed to occur. For the penultimate period $t = |T| - 1 = 5$, it is the sum of discounted future certain CFs *before* and uncertain CFs *after* the ultimate outstanding jump. Analogously, for $t < |T| - 1 = 5$, it is the sum of certain and uncertain CFs.

$$\overline{NPV}^{sc}_{t,p,d} = \begin{cases} \sum_{y=0}^{L_3-1} \rho_{p,y} CF^{sc}_{y,p,d} & \text{if } t = |T| \\ \rho_{p,(|T|-t)L_1} \sum_{sc'=1}^{SC} (g'^{sc,t}_{sc',|T|} \sum_{y=0}^{L_3-1} \rho_{p,y} CF^{sc'}_{y+L_1,p,d}) + \sum_{y=0}^{L_1-1} \rho_{p,y} CF^{sc}_{y,p,d} & \text{if } t = |T|-1 \\ \sum_{j=t+1}^{|T|-1} \rho_{p,(j-t)L_1} \sum_{sc'=1}^{SC} (g'^{sc,t}_{sc',j} \sum_{y=0}^{L_1-1} \rho_{p,y} CF^{sc'}_{y+jL_1,p,d}) + \rho_{p,L_2} \sum_{sc'=1}^{SC} (g'^{sc,t}_{sc',|T|} \sum_{y=0}^{L_3-1} \rho_{p,y} CF^{sc'}_{y+L_2,p,d}) + \sum_{y=0}^{L_1-1} \rho_{p,y} CF^{sc}_{y,p,d} & \text{if } t < |T|-1 \end{cases} \quad (2)$$

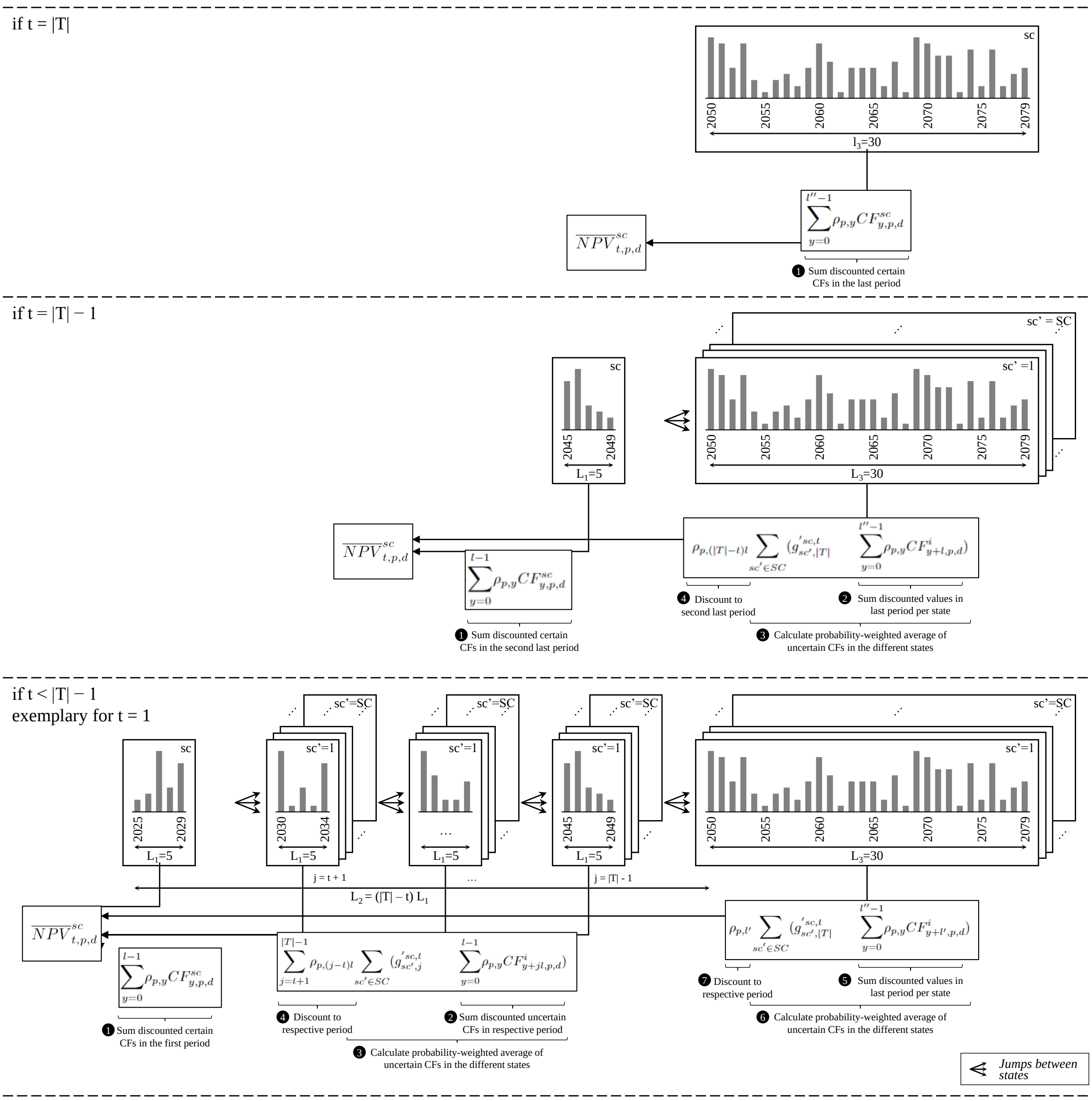

Supplementary Figure 6: **Net present value (NPV) calculation.** Calculation of $\overline{NPV}$ based on summing, discounting, and weighting of future cash flows.

The number of years between the decision in period t and the ultimate decision in period $|T|$ is defined as L_2 in Supplementary Equation (3) (between 0 and 25 years).

$$L_2 = (|T| - t) \times L_1 = (6 - t) \times 5 \tag{3}$$

The number of years modeled after the ultimate decision is defined as $L_3 = 30$ in Supplementary Equation (4).

$$L_3 = |Y| - (|T| - 1) \times L_1 = 55 - 5 \times 5 = 30 \tag{4}$$

The total number of years modeled is defined as $|Y| = 55$ in Supplementary Equation (5) as the total years between the end year $Y_{end} = 2079$ and start year $Y_{start} = 2025$.

$$|Y| = Y_{end} - Y_{start} = 2080 - 2025 = 55 \tag{5}$$

The discount factor $\rho_{p,y}$ is defined based on the plant-specific discount rate r_p (cf. Methods) and the number of years y to discount over as specified in Supplementary Equation (6).

$$\rho_{p,y} = \frac{1}{(1+r_p)^y} \tag{6}$$

It should be noted that including the three uncertainties explicitly through real options should influence the discount rate. This effect is not considered in the present article.

The probability of a jump from state sc in period t to state $sc' \in SC$ in any future period $j \in T$ is defined as $g'^{sc,t}_{sc',j}$ in Supplementary Equation (7). It is calculated as the sum of all possible trajectories' (referred to as *developments of the investment environment* in the Main) probabilities of jumping from sc in t to sc' in j (cf. Supplementary Figure 7 for a visualization and Section *States, uncertainties, and jump probabilities* for the definition of g and trajectory restrictions).

$$g'^{sc,t}_{sc',j} = \begin{cases} g^{sc}_{sc',t} & \text{if } j = t+1 \\ \sum_{sc_1=1}^{SC} g^{sc}_{sc_1,t}\, g^{sc_1}_{sc',t+1} & \text{if } j = t+2 \\ \sum_{sc_2=1}^{SC}\sum_{sc_1=1}^{SC} g^{sc}_{sc_1,t}\, g^{sc_1}_{sc_2,t+1}\, g^{sc_2}_{sc',t+2} & \text{if } j = t+3 \\ \sum_{sc_3=1}^{SC}\sum_{sc_2=1}^{SC}\sum_{sc_1=1}^{SC} g^{sc}_{sc_1,t}\, g^{sc_1}_{sc_2,t+1}\, g^{sc_2}_{sc_3,t+2}\, g^{sc_3}_{sc',t+3} & \text{if } j = t+4 \\ \sum_{sc_4=1}^{SC}\sum_{sc_3=1}^{SC}\sum_{sc_2=1}^{SC}\sum_{sc_1=1}^{SC} g^{sc}_{sc_1,t}\, g^{sc_1}_{sc_2,t+1}\, g^{sc_2}_{sc_3,t+2}\, g^{sc_3}_{sc_4,t+3}\, g^{sc_4}_{sc',t+4} & \text{if } j = t+5 \end{cases} \tag{7}$$

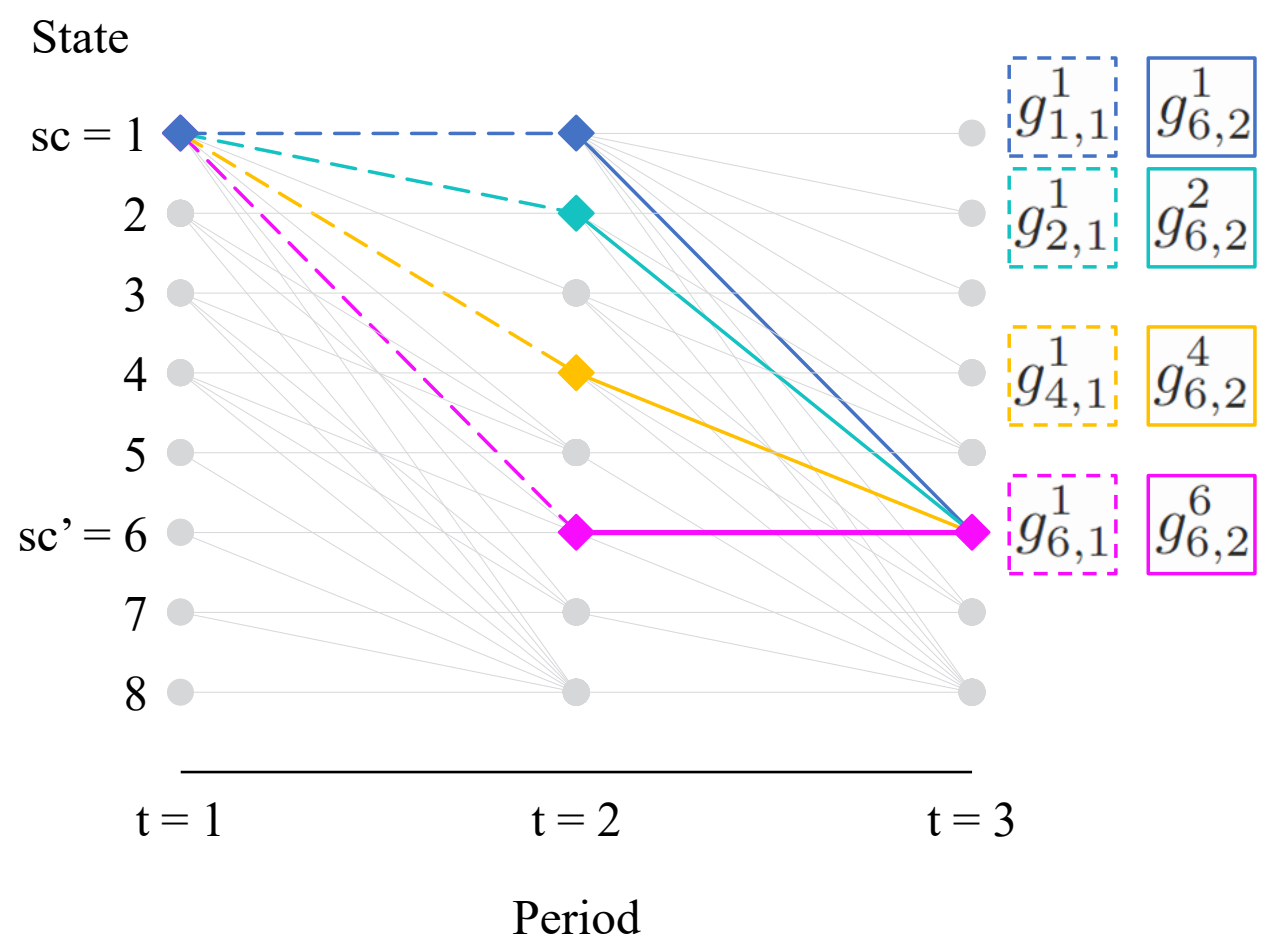

Supplementary Figure 7: **Visualization of an exemplary jump.** Exemplary jump probabilities for $g'^{1,1}_{6,3}$, i.e., a jump from state 1 in period 1 to state 6 in period 3

Payback criterion

Any available investment opportunity is only considered by a plant if its payback time meets a defined threshold c_p (= 15 years) as specified in Supplementary Equation (8) (payback criterion) where $\overline{NPV}'^{sc}_{t,p,d}$ is defined in Supplementary Equation (9) as the sum of discounted cash flows over c_p years. Accordingly, investment opportunities are only taken into account for the calculations of the option values if they meet the payback criterion. In comparison to $\overline{NPV}$ described in Supplementary Equation (2) $\overline{NPV}'$, which is used to test the payback criterion, is assumed not to consider uncertainty.

$$\overline{NPV}^{sc}_{t,p,d} = \begin{cases} \overline{NPV}^{sc}_{t,p,d} & \text{if } \overline{NPV}'^{sc}_{t,p,d}(c_p) \geq 0 \\ 0 & otherwise \end{cases} \tag{8}$$

$$\overline{NPV}'^{sc}_{t,p,d}(c_p) = \sum_{y=0}^{c_p-1} \rho_{p,y} CF^{sc}_{y,p,d} \tag{9}$$

NPV criterion

As plants are assumed to maximize their profits, the decision taken per period, plant, and state has the highest NPV of all possible investment opportunities $D_{t,p}$ as specified in Supplementary Equation (10) (NPV criterion)

$$NPV^{sc}_{t,p} = \max_{d_t \in D_{t,p}} NPV^{sc}_{t,p,d} \tag{10}$$

where the NPV is defined as the common $\overline{NPV}$ plus the option value $V^{sc}_{t,p,d}$ of taking decisions in the next period $t+1$ after having taken decision d in t as specified in Supplementary Equation (11).

$$NPV^{sc}_{t,p,d} = \overline{NPV}^{sc}_{t,p,d} + V^{sc}_{t,p,d} \tag{11}$$

Option values

The option value $V^{sc}_{t,p,d}$ is defined as zero for the ultimate period $t = |T|$ in which plants make decisions. For all $t < |T|$ it is defined as the state-weighted maximum of $\overline{NPV}$ of all investment opportunities $D_{t+1,p}$ in $t+1$ corrected for $corr^{sc}_{t,p}$ and discounted by the length of one period ρ_{p,L_1} as specified in Supplementary Equation (12)

$$V^{sc}_{t,p,d} = \begin{cases} \rho_{p,L_1} \sum_{sc'=1}^{SC} g^{sc}_{sc',t} \max_{d_{t+1} \in D_p(d_t)} (NPV^{sc'}_{t+1,p}(d_{t+1}) - corr^{sc}_{t,p}(d_t)) & \text{if } t < |T| \\ 0 & \text{if } t = |T| \end{cases} \tag{12}$$

with $corr^{sc}_{t,p}(d_t)$ being a term that corrects for the interdependence of staggered investments as defined in Supplementary Equation (13) by subtracting cash flows already included in $\overline{NPV}^{sc}_{t,p,d}$ of decision d_t taken in t

$$corr^{sc}_{t,p,d} = \begin{cases} \rho_{p,(|T|-t)L_1} \sum_{sc'=1}^{SC} ({g'}^{sc,t}_{sc',|T|} \sum_{y=0}^{L_3-1} \rho_{p,y} CF^{sc'}_{y+L_1,p,d}) & \text{if } t = |T|-1 \\ \sum_{j=t+1}^{|T|-1} \rho_{p,(j-t)L_1} \sum_{sc'=1}^{SC} ({g'}^{sc,t}_{sc',j} \sum_{y=0}^{L_1-1} \rho_{p,y} CF^{sc'}_{y+jL_1,p,d}) & \\ \quad + \rho_{p,L_2} \sum_{sc'=1}^{SC} ({g'}^{sc,t}_{sc',|T|} \sum_{y=0}^{L_3-1} \rho_{p,y} CF^{sc'}_{y+L_2,p,d}) & \text{if } t < |T|-1 \end{cases} \tag{13}$$

States, uncertainties, and jump probabilities

When the first decision is made in 2025, the model is, by definition, in state RRR (i.e., investment environment 1 in Fig. 1d in the Main). However, the probability of being in state RRR reduces with each period. The last investment decision will be taken in 2050 after the last jump between states. Accordingly, the last decision is not made under uncertainty.

Each state is defined by a combination of the three uncertainties (i.e., investment determinants) introduced (cf. Fig. 1 in the Main) with two vectors, a reference (R) vector and an alternative (A) vector: (1) a change in the carbon price, which changes the specific cost associated with emitting a ton of CO_2; (2) the buildout of CO_2 transport infrastructure, which change the specific CO_2 storage and transport (ST) costs; (3) a sudden reduction in capital expenditures (CAPEX) of CCS installations, which changes the specific capital expenditures.

Supplementary Table 6: Definition of states

States sc		Uncertainty n (i.e., uncertain investment determinant)		
No	Name	(1)	(2)	(3)
1	RRR	R: ETS PRICE ↑	R: ST COSTS ↑	R: CCS CAPEX ↑
2	RRA	R: ETS PRICE ↑	R: ST COSTS ↑	A: CCS CAPEX ↓
3	RAR	R: ETS PRICE ↑	A: ST COSTS ↓	R: CCS CAPEX ↑
4	RAA	R: ETS PRICE ↑	A: ST COSTS ↓	A: CCS CAPEX ↓
5	ARR	A: ETS PRICE ↓	R: ST COSTS ↑	R: CCS CAPEX ↑
6	ARA	A: ETS PRICE ↓	R: ST COSTS ↑	A: CCS CAPEX ↓
7	AAR	A: ETS PRICE ↓	A: ST COSTS ↓	R: CCS CAPEX ↑
8	AAA	A: ETS PRICE ↓	A: ST COSTS ↓	A: CCS CAPEX ↓

$g^{sc}_{sc',t}$, as used in Supplementary Equation (7) and Supplementary Equation (12), is the probability of a jump from state sc in t to state sc' in the subsequent period $t+1$ and defined as the product of the individual jump probabilities $\bar{g}^{sc}_{sc',t,n}$ $n \in N = 3$, which represent the probability of each of the individual uncertainties (i.e., investment determinants) to change vector as specified in Supplementary Equation (14). The individual jump probabilities $\bar{g}^{sc}_{sc',t,n}$ for each vector $n \in N$ and state $sc \in SC$ are defined in Supplementary Equations (15a), (15b), and (15c).

$$g^{sc}_{sc',t} = \prod_{n \in N} \bar{g}^{sc}_{sc',t,n} \tag{14}$$

$$\bar{g}^{sc}_{sc',t,1} = \begin{cases} 0 & \text{if } sc \in \{1,2,3,4\} \wedge \ sc' \in \{5,6,7,8\} \\ 1 & \text{if } sc \in \{1,2,3,4\} \wedge \ sc' \in \{1,2,3,4\} \\ \alpha_t & \text{if } sc \in \{5,6,7,8\} \wedge \ sc' \in \{1,2,3,4\} \\ \tilde{\alpha}_t & \text{if } sc \in \{5,6,7,8\} \wedge \ sc' \in \{5,6,7,8\} \end{cases} \tag{15a}$$

$$\bar{g}^{sc}_{sc',t,2} = \begin{cases} 0 & \text{if } sc \in \{1,2,5,6\} \wedge \ sc' \in \{3,4,7,8\} \\ 1 & \text{if } sc \in \{1,2,5,6\} \wedge \ sc' \in \{1,2,5,6\} \\ \beta_t & \text{if } sc \in \{3,4,7,8\} \wedge \ sc' \in \{1,2,5,6\} \\ \tilde{\beta}_t & \text{if } sc \in \{3,4,7,8\} \wedge \ sc' \in \{3,4,7,8\} \end{cases} \tag{15b}$$

$$\bar{g}^{sc}_{sc',t,3} = \begin{cases} 0 & \text{if } sc \in \{1,3,5,7\} \wedge \ sc' \in \{2,4,6,8\} \\ 1 & \text{if } sc \in \{1,3,5,7\} \wedge \ sc' \in \{1,3,5,7\} \\ \gamma_t & \text{if } sc \in \{2,4,6,8\} \wedge \ sc' \in \{1,3,5,7\} \\ \tilde{\gamma}_t & \text{if } sc \in \{2,4,6,8\} \wedge \ sc' \in \{2,4,6,8\} \end{cases} \tag{15c}$$

with α_t, β_t, and γ_t being defined as the probability to jump from the *reference* to an *alternative* vector between in period t for uncertainty 1, 2 and, 3, respectively. Accordingly, the probabilities of staying at the reference vector, i.e., $\tilde{\alpha}_t$, $\tilde{\beta}_t$, and $\tilde{\gamma}_t$, are defined as $1-\alpha$, $1-\beta$, and $1-\gamma$, respectively. Only uni-directional changes are assumed, i.e., uncertain investment determinants can only jump from the reference to the alternative vector and not vice versa, and the probability of a jump from the alternative to the reference vector is accordingly always zero and, as a consequence, that of staying at the alternative vector always one. Combining the individual jump probabilities results in the combined jump probabilities shown in Supplementary Table 7.

Supplementary Table 7: Combined jump probabilities for period t

Jump from state sc...	Jump to state sc' ...							
	8	7	6	5	4	3	2	1
8	1	0	0	0	0	0	0	0
7	γ_t	$\tilde{\gamma}_t$	0	0	0	0	0	0
6	β_t	0	$\tilde{\beta}_t$	0	0	0	0	0
5	$\beta_t\gamma_t$	$\beta_t\tilde{\gamma}_t$	$\tilde{\beta}_t\gamma_t$	$\tilde{\beta}_t\tilde{\gamma}_t$	0	0	0	0
4	α_t	0	0	0	$\tilde{\alpha}_t$	0	0	0
3	$\alpha_t \gamma_t$	$\alpha_t \tilde{\gamma}_t$	0	0	$\tilde{\alpha}_t \gamma_t$	$\tilde{\alpha}_t \tilde{\gamma}_t$	0	0
2	$\alpha_t\beta_t$	$\alpha_t\beta_t$	$\alpha_t\tilde{\beta}_t$	0	$\tilde{\alpha}_t\beta_t$	0	$\tilde{\alpha}_t\tilde{\beta}_t$	0
1	$\alpha_t\beta_t\gamma_t$	$\alpha_t\beta_t\tilde{\gamma}_t$	$\alpha_t\tilde{\beta}_t\gamma_t$	$\alpha_t\tilde{\beta}_t\tilde{\gamma}_t$	$\tilde{\alpha}_t\beta_t\gamma_t$	$\tilde{\alpha}_t\beta_t\tilde{\gamma}_t$	$\tilde{\alpha}_t\tilde{\beta}_t\gamma_t$	$\tilde{\alpha}_t\tilde{\beta}_t\tilde{\gamma}_t$

Using Supplementary Equations (7), (14), and (15a)-(15c) as well as the jump probabilities given in Supplementary Table 3 allows to calculate the state probabilities at different points in time as shown in Supplementary Tables 8 and 9 for the NZ2050 and Delayed scenario, respectively.

Supplementary Table 8: State probabilities for NZ2050 scenario

	Period	1	2	3	4	5	6
	Years	2025-2029	2030-2034	2035-2039	2040-2044	2045-2049	2050-2079
State sc							
No	Name						
1	RRR	100	24.2	0.6	0.0	0.0	0.0
2	RRA	0	23.7	6.2	0.3	0.2	0.1
3	RAR	0	1.3	0.2	0.0	0.0	0.0
4	RAA	0	1.2	1.6	0.1	0.2	0.2
5	ARR	0	23.7	6.2	1.0	0.0	0.0
6	ARA	0	23.3	66.3	65.0	55.1	46.0
7	AAR	0	1.2	1.6	0.5	0.0	0.0
8	AAA	0	1.2	17.3	33.1	44.5	53.7

Supplementary Table 9: State probabilities for Delayed scenario

	Period	1	2	3	4	5	6
	Years	2025-2029	2030-2034	2035-2039	2040-2044	2045-2049	2050-2079
State sc							
No	Name						
1	RRR	100	47.7	21.8	7.7	2.7	1.6
2	RRA	0	0.2	1.3	2.0	1.4	1.3
3	RAR	0	46.8	63.6	51.8	38.8	27.3
4	RAA	0	0.2	3.7	13.8	20.1	22.3
5	ARR	0	2.5	2.4	2.5	1.6	1.4
6	ARA	0	0.0	0.1	0.7	0.8	1.2
7	AAR	0	2.5	6.9	16.9	22.8	24.7
8	AAA	0	0.0	0.4	4.5	11.8	20.2

In Supplementary Equation (12) $D_p(d_t)$ denotes the available decisions in period $t+1$ if decision d was taken in period t. If a plant chose to WAIT ($d = 1$) in period t, it could still pursue decisions 1–4 in period $t + 1$. If a plant pursued the decision CCS in period t ($d = 2$) or any period before ($d = 7$), it can still do BIO ($d = 6$) or wait ($d = 7$) in period $t + 1$. Whereas if a plant took the decision BIO in period t ($d = 3$) or any period before ($d = 8$), it can still do CCS ($d = 5$) or wait ($d = 8$) in period $t + 1$. After CCS and BIO ($d = [4, 6]$), however, no additional decisions are assumed to be available. Supplementary Equation (16) and Supplementary Figure 8 summarize a plant's options space.

$$D_p(d_t) = \begin{cases} [1,4] & \text{if } d_t = 1 \\ \{6,7\} & \text{if } d_t \in \{2,7\} \\ \{5,8\} & \text{if } d_t \in \{3,8\} \\ \{\} & \text{if } d_t \in [4,6] \end{cases} \tag{16}$$

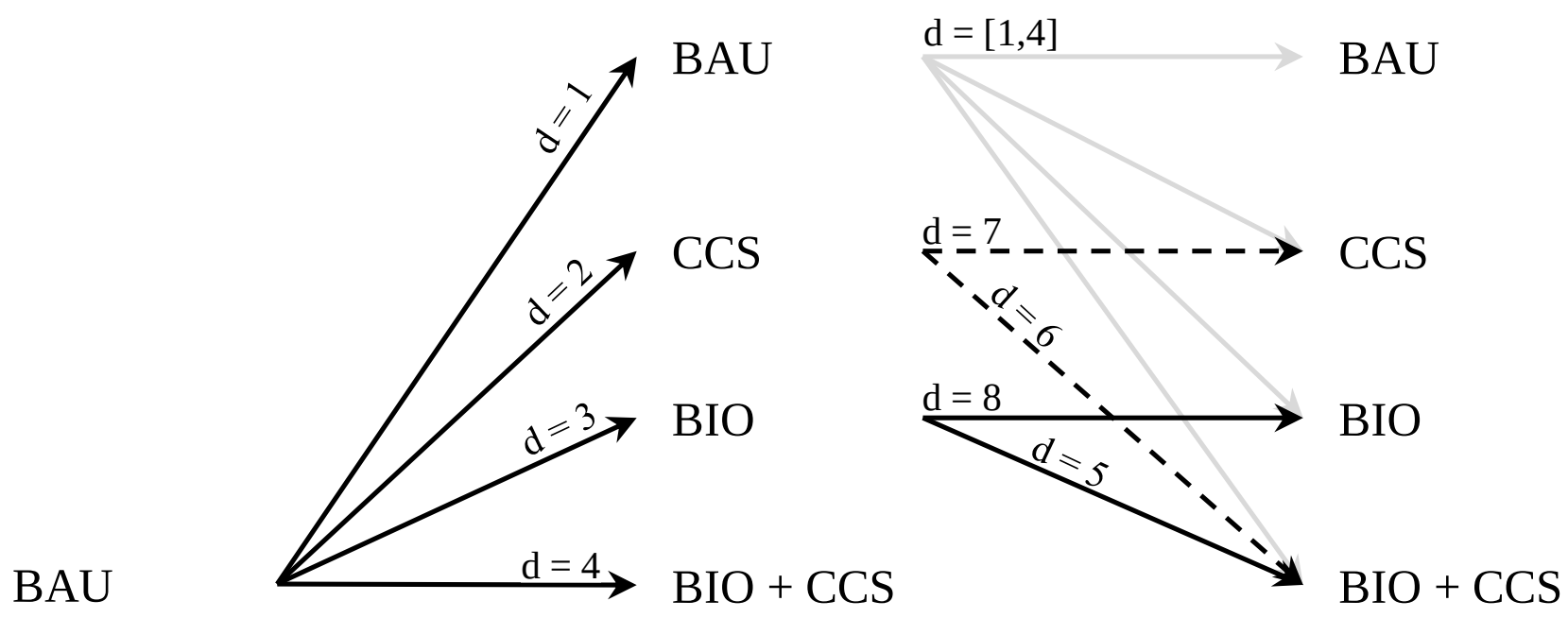

Supplementary Figure 8: **Option space for a representative plant.** Option space for investment decisions of a representative plant in the real options valuation model. Future options are contingent on decisions in previous periods.

Cash flow and cost components

The cash flow $CF^{sc}_{y,p,d}$ per year used to calculate the NPVs is defined in Supplementary Equation (17) as the difference between relative ETS savings $\Delta S^{sc}_{y,p,d}$ and relative costs $\Delta C^{sc}_{y,p,d}$ with savings and costs being defined relative to a business as usual case that assumes existing cement kilns are operated using the same fuel mixes. No additional benefits are considered, e.g., green premiums on the sales price of the goods produced.

$$CF^{sc}_{y,p,d} = \Delta S^{sc}_{y,p,d} - \Delta C^{sc}_{y,p,d} \tag{17}$$

The relative savings per year are defined in Supplementary Equation (18) as the product of the difference in absolute emissions $\Delta E^{foss}_{y,p,d}$ and the applied carbon price $b^{sc}_{y,p,d}$

$$\Delta S^{sc}_{y,p,d} = \Delta E^{foss}_{y,p,d}\, b^{sc}_{y,p,d} \tag{18}$$

with the difference in absolute emissions being defined in Supplementary Equation (19) as the product of the difference in specific emissions $\Delta e^{foss}_{y,p,d}$ and the plant size represented by the yearly heat demand h_p

$$\Delta E^{foss}_{y,p,d} = h_p\, \Delta e^{foss}_{y,p,d} \tag{19}$$

and where $\Delta e^{foss}_{y,p,d}$ is defined in Supplementary Equation (20) as the sum of process- and fuel-related emissions $\Delta e^{proc}_{y,p,d}$ and $\Delta e^{fuel}_{y,p,d}$, respectively, where the latter is defined in Supplementary Equation (21) as the sum of the fuel constituents' $f \in F_d$ net emission factors ef^{net}_f weighted by their share per decision $\Delta s_{f,d}$ (relative to the incumbent technology) and corrected for the efficiencies of the constituents $\eta_{f,d}$. As opposed to the ef^{gr}_f, which represents absolute emissions (i.e., the sum of biogenic and fossil emissions), ef^{net}_f indicates only fossil emissions.

$$\Delta e^{foss}_{y,p,d} = \Delta e^{proc}_{y,p,d} + \Delta e^{fuel}_{y,p,d} \tag{20}$$

$$\Delta e^{fuel}_{y,p,d} = \sum_{f \in F_d} \frac{ef^{net}_f}{\eta_{f,d}} \Delta s_{f,y,p,d} \tag{21}$$

Relative yearly costs are defined in Supplementary Equation (22) as the sum of investment (ΔI), fuel (ΔFU), operations & maintenance (ΔOM), and CO_2 storage & transport (ΔST) costs relative to the incumbent setup.

$$\Delta C^{sc}_{y,p,d} = \Delta I^{sc}_{y,p,d} + \Delta FU^{sc}_{y,p,d} + \Delta OM^{sc}_{y,p,d} + \Delta ST^{sc}_{y,p,d} \tag{22}$$

The components can be broken down further using Supplementary Equations (23)-(26).

$$\Delta I^{sc}_{y,p,d} = H_p \; \Delta i^{sc}_{y,p,d} \tag{23}$$

$$\Delta FU^{sc}_{y,p,d} = \frac{h_p}{\eta_{f,d}} \Delta fu^{sc}_{y,p,d} \tag{24}$$

$$\Delta OM_{y,p,d} = H_p \; \Delta om_{y,p,d} \tag{25}$$

$$\Delta ST_{y,p,d} = \Delta E_{capt_{y,p,d}} \Delta st^{sc}_{y,p,d} \tag{26}$$

where IF_d represents an integration factor, H_p is defined in Supplementary Equation (27) as a plant's yearly heat demand h_p divided by the plant's annual load hours FL_p corrected for an efficiency penalty pen to account for a surplus in energy demand due to CCS, and where $\Delta E_{capt_{y,p,d}}$ is the amount of CO_2 captured that needs to be transported and stored as defined in Supplementary Equation (28).

$$H_p = \frac{h_p}{FL_p} pen \tag{27}$$

$$\Delta E_{capt_{y,p,d}} = \begin{cases} H_p CR \sum_{f \in F_d} \frac{ef^{gr}_f}{\eta_{f,d}} \Delta s_{f,y,p,d} & \text{if } d \in \{2,4,5,6,7\} \\ 0 & \text{else} \end{cases} \tag{28}$$

For CCS, an installation period of five years is assumed, and for BIO, one of two years. The CAPEX are equally spread over the installation period. The OPEX (FU, OM, and ST) are only considered after the end of the installation period. When evaluating the payback criterion, one has to consider that the first year of the installation period is used as a reference for the payback period. Depending on the assumed service life of the decarbonization technologies, reinvestments are considered at the end of service life.

Supplementary Notes

Supplementary Note 1: Sensitivitiy analysis on plant age

Our results raise the question how plants of different age would invest in the uncertain investment environment we model. While our dataset does not hold the granularity to take plant-specific age data into account, we can emulate the effect by varying the initial CAPEX investment. This is because investment decisions are taken relative to re-investment into the conventional plant. Consequently, for older plants re-investment would be necessary in any case, and the relative CAPEX of a low-carbon investment would be lower than in the base case. For newer plants, in turn, investors would have to take the remaining depreciation value of the conventional facility into account. The resulting sunk cost would have to be reflected in the new investment, which we represent by an increase in (relative) CAPEX. We consequently approximate a fleet of older plants with a 25% CAPEX reduction, and one of newer plants with a 25% CAPEX increase. We are aware that this is a stylized assumption, and the sensitivity analysis below demonstrates the direction and approximate magnitude of the effect rather than a precise calibration.

Investment behavior of older plants

We find that the NZ2050 scenario results are robust with respect to higher plant age as approximated with a reduction in effective CAPEX. In the Delayed scenario, however, when confidence in future carbon price levels and CO_2 transport infrastructure is low, a portfolio of older plants leads to a modestly higher share of low-carbon investment. This is shown by Supplementary Figure 9, which replicates Figure 2 of the manuscript with the assumed CAPEX reduction. It shows that higher plant age accelerates the transition slightly and lowers the long-run equilibrium share of plants that do nothing (BAU) to 12% compared to the Delayed scenario with our baseline CAPEX assumptions, where 17% do not invest. Consequently, an older initial stock of cement plants in the Delayed scenario leads to lower yearly emissions in the mid to long term, as well as reduces cumulative cement emissions between 2025 and 2050 by 0.1 $GtCO_2$, to 0.7 Gt in total.

Investment behavior of newer plants

We find that the NZ2050 scenario results are equally robust with respect to lower plant age, and the results remain unchanged relative to our baseline CAPEX assumptions. In the Delayed scenario, however, a portfolio of newer plants slows the transition, as the higher effective CAPEX – reflecting the sunk cost of remaining depreciation – raises the threshold for low-carbon investment under uncertainty. This is depicted in Supplementary Figure 10, which shows that lower plant age raises the long-run equilibrium share of plants that remain uninvested to 26% (compared to 17% in the Delayed scenario with our baseline CAPEX assumptions). As a consequence, cumulative emissions from the cement sector between 2025 and 2050 increase by 0.2 $GtCO_2$ relative to our baseline CAPEX, resulting in 1 Gt in total.

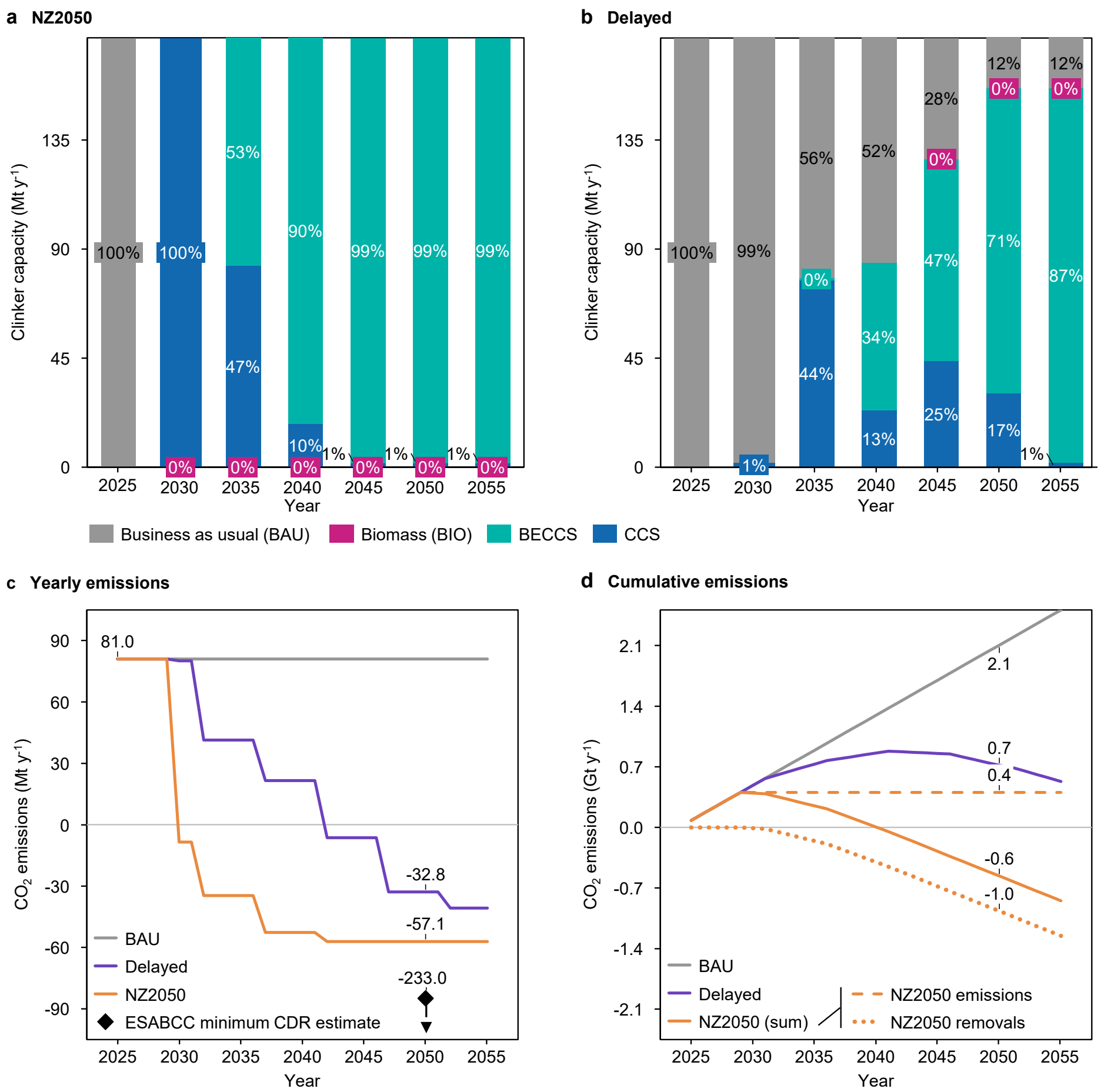

Supplementary Figure 9: **Sensitivity analysis with older plant fleet.** Technology-specific deployment patterns and respective emissions outcomes with 25% lower effective capital expenditure (CAPEX). **a, b**, Deployment of biomass, carbon capture and storage (CCS), as well as their combination (BECCS) in the NZ2050 and Delayed scenarios, respectively. **c, d**, Yearly emissions (c) in relation to the minimum estimate for Carbon Dioxide Removal (CDR) by the European Scientific Advisory Board on Climate Change (ESABCC), and cumulative emissions (d).

From the sensitivity analysis, we conclude that plant age has a larger influence when confidence in the low-carbon investment environment is low. Both for an older and a newer plant fleet, our Delayed scenario reacts to the change in relative CAPEX. More specifically, an initial set of older plants increases the pace of the transition, and vice versa. In the NZ2050 scenario, on the other hand, confidence in the investment environment results in a business case that is practically independent from variations in effective CAPEX in the assumed order of magnitude. We conclude that, in the investment environment of the NZ2050 scenario, plant age would only play a minor role.

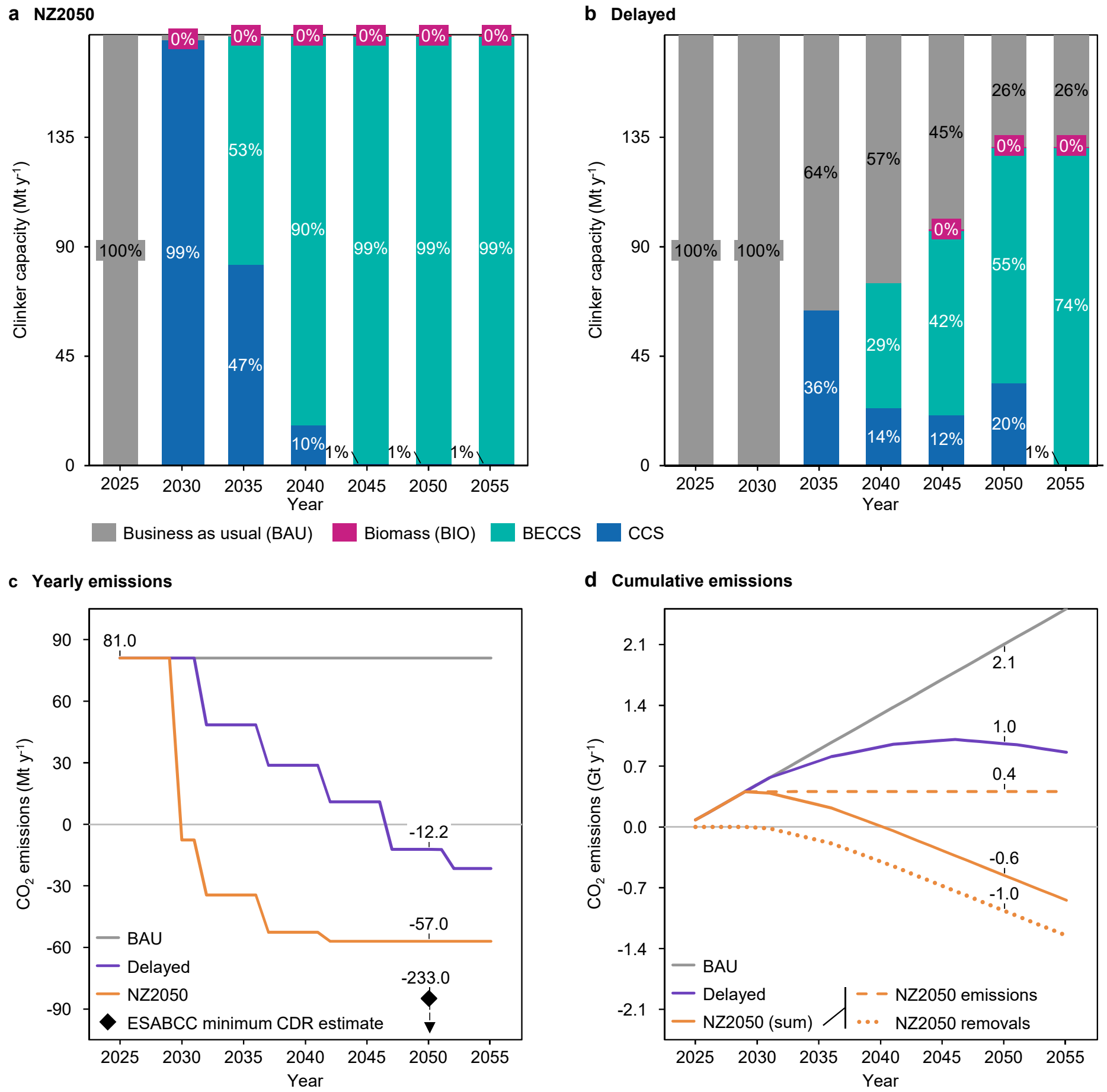

Supplementary Figure 10: **Sensitivity analysis with newer plant fleet.** Technology-specific deployment patterns and respective emissions outcomes with 25% higher effective capital expenditure (CAPEX). **a, b**, Deployment of biomass, carbon capture and storage (CCS), as well as their combination (BECCS) in the NZ2050 and Delayed scenarios, respectively. **c, d**, Yearly emissions (c) in relation to the minimum estimate for Carbon Dioxide Removal (CDR) by the European Scientific Advisory Board on Climate Change (ESABCC), and cumulative emissions (d).

Supplementary References

[1] Michael Pahle et al. "The emerging endgame: The EU ETS on the road towards climate neutrality". In: *Resource and Energy Economics* 81 (Feb. 2025), p. 101476. ISSN: 0928-7655. DOI: 10.1016/J.RESENEECO.2024.101476.

[2] European Cement Research Academy. *The ECRA Technology Papers 2022 - State of the Art Cement Manufacturing - Current Technologies and their Future Development*. Tech. rep. Duesseldorf, 2022. URL: https://ecra-online.org/research/technology-papers.

[3] Cemsuisse. *Kennzahlen*. Tech. rep. Bern: Verband der Schweizerischen Cementindustrie, Jan. 2021.

[4] Carbon Limits and CATF. *The cost of carbon capture and storage in Europe*. 2022. URL: https://www.catf.us/ccs-cost-tool/.

[5] P. Ruiz et al. "ENSPRESO - an open, EU-28 wide, transparent and coherent database of wind, solar and biomass energy potentials". In: *Energy Strategy Reviews* 26 (Nov. 2019), p. 100379. ISSN: 2211-467X. DOI: 10.1016/J.ESR.2019.100379.

[6] Paul Tautorat et al. "Real options analysis of decarbonization investments in the chemical industry". In: *Applied Energy* (2025). DOI: 10.1016/j.apenergy.2025.126238. URL: https://doi.org/10.1016/j.apenergy.2025.126238.
