# Supplementary material for: Decarbonizing the EU cement industry under technology and policy uncertainty
Source: Nat Commun. 2026 Aug 17;17:9862. doi: 10.1038/s41467-026-76611-3 (PMC13578682; doi:10.1038/s41467-026-76611-3)
Supplement: Supplementary file 2 [file 41467_2026_76611_MOESM2_ESM.pdf]

nature portfolio

Peer Review File

Decarbonizing the EU cement industry under technology and policy uncertainty

Corresponding Author: Professor Tobias Schmidt

This file contains all reviewer reports in order by version, followed by all author rebuttals in order by version.

Version 0:

Reviewer comments:

Reviewer #1

(Remarks to the Author)
Review manuscript number NCOMMS-25-79318-T
1. General comment
This is a concise study with an innovative methodological approach, making effective use of publicly available data. The main results highlighted by the authors are:
1. A delayed transition of the decarbonization of the cement industry occurs under conditions of low confidence and low stringency in the EU ETS.
2. Cement plants with good access to CO2 transport infrastructure (coastal or waterway proximity) are more likely to transition earlier.
3. Biomass plays a major role in the transition of the cement industry and provides potential for carbon dioxide removal (CDR).
While the first two findings largely confirm previous work (see e.g. Emanuelsson et al., 2025; Favier et al., 2018; Obrist et al., 2021; and Rootzén and Johnsson, 2011), the third finding represents a novel point of view and potentially significant contribution. However, the treatment and representation of biomass should be further strengthened to increase the credibility of the paper through clearer articulation of key assumptions and supporting evidence (see more detailed comments on this below).
2. Significance and relation to existing literature
The work is of clear relevance to the decarbonization of the cement sector and related industrial transitions. The methodological innovation, particularly the integration of expert elicitation with site-level techno-economic modelling, adds value to the field. However, the results largely reinforce established insights (e.g., the influence of ETS policy design and infrastructure access), and the connection to existing literature could be better developed through more explicit referencing, discussion and comparison.
Overall, the contribution lies more in the methodological framework than in the novelty of the findings.
3. Support for conclusions and claims
The conclusions are generally supported by the presented results, but additional evidence and clarification would improve their robustness. In particular, assumptions around biomass availability, cost development over time, and process-specific limitations should be made more transparent. The authors appropriately reference the respective data sources from which the data were collected, but without additional information (e.g., in the supplementary material), it is difficult for the reader to understand how the data have been used and how different assumptions influence the result. We also don't find a proper description of what type of biomass you assume to be available, you mention agricultural biomass, but when looking at Figure 4 and the potential for Sweden we assume that you also include forestry biomass. Further, the distinction between how much biomass is currently used in the industry (in the BAU scenario since all fuel today are not of fossil origin) and future use (BIO scenario) could be clarified. Specifically in relation to Figure 2a and 2b.
Further detail on site-specific cost assumptions (OPEX, CAPEX, transport and storage costs) would strengthen credibility. We for example find that the OPEX assumptions are not well-listed in the paper, does all plants have the same OPEX or do they differ, and in such case how and why?
We also greatly miss a discussion on the technology choices in the paper (see e.g. Cavalett which is already referenced in the paper). The authors does not acknowledge potential abatement options beyond CCS, fuel switching from fossil to biomass, and the combination of those two (BECCS). We think it is important to highlight that decarbonization needs to occur along the full value chain and that there are more options to decarbonization than just adding an end-of-pipe type of solution such as CCS (even though we agree that CCS is a highly important mitigation option in the cement industry) (see

e.g. Habert et al., 2020). But there are/will be other mitigation options available such as electrification to remove the fuel emissions (which would then cancel out the whole CDR part of this paper), demand reduction through Supplementary Cementitious Materials (SCMs), demand side reduction, and so on. Again, we strongly agree that CCS is a main mitigation option for the cement industry but this exclusion of other mitigation options should be justified and proper motivated in the paper.

4. Data analysis, interpretation, and conclusions

The analysis appears technically sound and the data treatment is carefully executed. However, some assumptions, particularly those affecting investment behaviour, require clearer justification. For instance:

- How do firms form expectations about ETS prices, CCS costs, and biomass availability?
- Do they have perfect foresight or limited information?
- How are market uncertainties (e.g., demand reduction, clinker substitution, sufficiency measures) represented?

Addressing these points would enhance the credibility of the behavioural dimension of the model.

The paper could also benefit from a more explicit discussion of the model's limitations, including factors outside plant owners' control (e.g., infrastructure buildout, policy readiness, or bilateral agreements for CO2 transport).

5. Methodology and standards

The methodology is innovative and meets high scientific standards. The integration of expert elicitation is a welcome feature and enhances the credibility of input assumptions. However, certain aspects could be elaborated to improve reproducibility and accessibility:

- Clarify cost assumptions (FOAK vs. NOAK, learning rates). You are showing how the investment cost is assumed to reduce over time in Figure 2c, but we did not find any stated assumptions about learning rates assumed to achieve those curves.
- Specify how transport infrastructure development is treated and who makes related investment decisions. Again, you show the input you regarding the transport costs use in the modelling in Figure 2b, but the assumptions made need to be clarified. What transport modes do you allow for (road, rail, barge, pipeline)? You consistently mention "available infrastructure" but what do you actually mean by that (physically in place? That it can take more load? Or something else?)? And again, is that infrastructure "availability" expanding over time, and is such case how and who takes that decision/investment?
- Provide more detail on how site-specific costs are calculated and applied. In the methodology you mention "kiln fuel mixes, biomass substitution, and thermal efficiency", are they assumed the same for all plants or different? And what are your assumptions?

While the mathematical formulation is thorough, the description is rather dense in the supplementary material. Simplifying or explaining key model components in more intuitive terms would make the paper more accessible to a broader audience. Currently, the methodological details are spread out in the main section, methodology, and supplementary material, which made it quite difficult to get a proper grasp of the full methodology even after reading everything. Our recommendation is to ask a colleague of yours that is not familiar with the methodology to read the paper to give feedback on how to improve the methodological description. Again, we think that it has thorough mathematical formulations but that you could appeal to a much broader audience by explaining it in more intuitive ways. But we also want to acknowledge that you have attempted to do this through several methodological illustrations (for example Figure 2e, and in the supplementary material).

6. Reproducibility and transparency

The study provides a relatively detailed methodological description, but some critical assumptions remain implicit. Providing more explicit information on data sources, parameter ranges, and decision rules would enhance reproducibility (as previously mentioned). References to all data sources should be complete and include publication details to facilitate verification by readers.

7. Recommendation

Overall, this is a high-quality and promising manuscript. The study is well executed and presents a sophisticated methodological contribution. However, given the need for clearer justification of assumptions, improved transparency of key parameters, and stronger linkage to existing literature, we recommend that the editor invite the authors to revise their manuscript before a final decision is made

8. Specific comments

Please note that while we have provided a large number of comments, these are intended as suggestions for further improvement rather than requirements. The manuscript is already strong, and not all points necessarily need to be addressed in detail.

Abstract

1. In your abstract you state that 0.8 Gt of CO2 emissions would remain by 2050. I think it helps the reader if you also state the emissions before the transition, to get a grasp of actual reductions.

Methodology

2. The Finnsementti plant in Lappeenranta seems to be missing in the data? It could be categorized according to the old activity codes in the ETS data (activity code 6 I believe).

Main

3. Statement: Line 23 "Combining CCS with a fuel switch to biogenic energy (BECCS) could permanently remove CO2 from the atmosphere.". Comment: even though you say "could", it would be beneficial to state here under what circumstances this applies.

4. Statement: Line 24-25 "Therefore, cement production could become an important contributor to carbon dioxide removal (CDR)". Comment: it would help the reader if you could also state some potential here for how much CDR the cement industry could actually contribute with in relation to what's needed. Then the reader could get an insight into whether the cement industry is an important player here or not.

5. Statement: Line 28-29 "Until then, the allowance cap will continue to gradually decline every year, making EU carbon pricing increasingly onerous.". Comment: "onerous" from an industrial actors point of view?

6. Statement: Line 31 "and over €100 billion in financing". Comment: and how much is that in comparison to what is needed

in the cement industry?
7. Statement: Line 33: “For the cement industry, uncertainty is particularly high:”. Comment: compared to what? Many industries are facing similar issues.
8. Statement: Line 42: “First, given the landscape of cement plants today, it remains unclear to what extent the sector overall can contribute to mitigating climate change, both through emissions reductions and the potential to deliver CDR.”. Comment: but how much they can reduce their emissions isn’t really unclear? Or do you mean cumulative emissions?
9. Statement: Line 50 “Second, more clarity is needed on the distributional implications of a “new economic geography of decarbonization” in the European cement industry.”. Comment: what do you mean by new economic geography of decarbonization? Perhaps good to define it?
10. Statement: Line 56 “there is limited understanding of how accounting for decision-making under uncertainty influences the geographical aspects of the cement industry’s decarbonization”. Comment: with “geographical aspects” do you mean how the location of the cement plants would be impacted? Or could you define what you mean here since you don’t discuss relocation in the paper.
11. You often say European cement industry but you are really looking at EU plus Switzerland.
12. By excluding non-integrated plants, how much of the total industry emissions are you neglecting?
13. Statement: Line 68 “This approach enables us to estimate the value of waiting, and to identify the effect of key uncertainties on timelines for decarbonization investments in the EU cement industry, including geographical patterns and variations in producers’ expectations about the future development of key investment determinants.”. Comment: what do you mean with “the value of waiting”? And I am not sure what you mean with “geographical patterns” here. Please clarify.

Investment patterns and emissions trajectories
14. Statement: Line 102 “In this “best-case” scenario”. Comment: best from what perspective? Better to call it “low-emission scenario” or other?
15. Swap places of Figure 2c and 2d since you explain 2d first in the text?
16. Is Figure 2b only transport cost or does it also include the cost for storage? Or have you stated that cost assumption elsewhere?
17. Figure 1c shows the specific CCS investment cost and the y-axis states that the unit is in M€ while the caption says M€ per ton of CO2 captured per hour. It could also be beneficial to the manuscript to mention this in absolute numbers (for example X M€ for a cement plant with a capacity of 1 Mt (or similar)), so that the reader doesn’t have to go to supplementary materials to check the order of magnitude.
Investment by country and plant
18. In the caption of Figure 3 you state: “BIO investments without CCS overall make up less than 1% (cf. Fig. 2) and are therefore not shown.”. But in Figure 2 BIO is always zero, is it rounded to zero?
Biomass demand for cement decarbonization
19. Statement: Line 193 “Cement decarbonization may use a substantial portion of Europe’s locally available biomass, depending on the country”. Comment: I think you need to add a discussion if this is actually an efficient use of biomass resources or if it would be more beneficial (from a climate perspective) to use these green carbon atoms in other sectors. Since this is a key part of the results this needs to be put into context and problematized more clearly. e.g. what is the cement industries “willingness to pay” for biomass compared other sectors in a carbon constrained future. What does the literature say about other uses of biomass and “green” C atoms?
20. Statement: Line 208 “While the cement industry will inevitably compete for this biomass with other sectors, cement producers have the advantage of being able to handle different types of (low-quality) biomass”. Comment: will they actually be able to compete for this biomass though? As you state in your introduction: “Cement production is particularly affected by increasing ETS allowance prices caused by the cap reduction, due to cement’s high emissions intensity relative to its low general cost”, the cement industry may not have much purchasing power to compete with other industries for this biomass. (See also comment above)
Discussion
21. Statement: “Second, clearer political guidance is needed on the role of cement in delivering negative emissions”. Comment: but I still think it’s very unclear (from a climate perspective) what role the cement industry should take in a negative emissions perspective since we don’t know yet where the biomass will be of “best use” from a climate perspective.

Suggested references

Emanuelsson, A. H., Rootzén, J., & Johnsson, F. (2025). Deployment of carbon capture and storage in the cement industry – Is the European Union up to shape? International Journal of Greenhouse Gas Control, 146, 104442. https://doi.org/10.1016/j.ijggc.2025.104442
Favier, A., De Wolf, C., Scrivener, K., & Habert, G. (2018). A sustainable future for the European Cement and Concrete Industry: Technology assessment for full decarbonisation of the industry by 2050. ETH Zurich. https://doi.org/10.3929/ethz-b-000301843
Habert, G., Miller, S. A., John, V. M., Provis, J. L., Favier, A., Horvath, A., & Scrivener, K. L. (2020). Environmental impacts and decarbonization strategies in the cement and concrete industries. Nature Reviews Earth & Environment, 1(11), 559–573. https://doi.org/10.1038/s43017-020-0093-3
Obrist, M. D., Hischier, R., & Horvath, A. (2021). Decarbonisation pathways of the Swiss cement industry to 2050: Long-term energy consumption and CO_2 emission scenarios. Journal of Cleaner Production, 298, 126746. https://doi.org/10.1016/j.jclepro.2021.126746
Rootzén, J., Kjärstad, J., & Johnsson, F. (2011). Prospects for CO2 capture in European industry. Management of Environmental Quality: An International Journal, 22(1), 18–32. https://doi.org/10.1108/14777831111098453

(Remarks on code availability)

Reviewer #2

(Remarks to the Author)
I co-reviewed this manuscript with one of the reviewers who provided the listed reports. This is part of the Nature Communications initiative to facilitate training in peer review and to provide appropriate recognition for Early Career Researchers who co-review manuscripts.

(Remarks on code availability)

Reviewer #3

(Remarks to the Author)
Decarbonizing the EU cement industry under technology and policy uncertainty
Review Comments
December 4, 2025

The paper models the effects of uncertainties related to ETS allowance prices, carbon-capture investment costs, and the availability of CO2 transport infrastructure affect decisions by cement plants across Europe to deploy carbon capture and storage and biomass use to reduce emissions and potentially turn the cement industry into a carbon sink.

I have two substantial comments.

The first relates to the impact of not considering the age of the plants and kilns. This appears to be due to a lack of plant-specific data. It seems likely, however, that a plant whose kiln needs to be replaced within 5 years would attach different weights to the uncertainties than a plant whose kiln has a projected lifespan of 20 years. If age/remaining lifespan data is not available, I suggest sensitivity analyses for a representative plant(s) assuming different ages to assess the impact on investment decisions.

I suspect that the effect would be accelerated adoption of CCS for plants with a remaining life of a decade or less and slower adoption for newer plants. Given the high rate of adoption of CCS as the initial mitigation option especially for the NZ2050 scenario, the results may be very similar even after adjusting for the age of the plant/kiln. Regardless I believe plant/kiln age should be explicitly addressed in some way.

The second relates to the emphasis on the ETS in the short section on policy implications. The paper asserts that “the EU needs to uphold and strengthen the credibility of its carbon market, … reach consensus … on a clear target picture for the time after an “ETS endgame, [and] … support the ETS credibly through the implementation of the Carbon Border Adjustment Mechanism (CBAM).”

Cement industry emissions account for less than 10% of the emissions covered by the ETS. An ETS provides an incentive for all covered sources to implement reductions whose marginal cost is less than the allowance price. Emission reductions through cement plant CCS are likely to be costly relative to the mitigation options available to other ETS participants. Thus relying on the ETS price to drive cement industry CCS adoption is likely to have limited impact until the lower cost options available to other participants are exhausted.

To me the message from the modelling results is clear – CCS is the first step toward cement industry emissions reductions and implementation of this technology can begin immediately. The policy implications, then, are equally clear; policies that stimulate early adoption of CCS by cement plants. The paper mentions two such policies -- Carbon Contracts for Difference (CCfDs) and a coherent long-term strategy for CO2 infrastructure – but buries them in the middle of the ETS discussion. Additional policies could include regulations that require new cement plants to install CCS and, probably with a lag, policies to increase the supply of biomass.

Revise the policy discussion to draw on the modelling results.

(Remarks on code availability)

Version 1:

Reviewer comments:

Reviewer #1

(Remarks to the Author)
Thank you for the opportunity to review the revised manuscript. We believe the authors have addressed the comments and questions thoroughly and have improved the manuscript accordingly. In our view, the manuscript is now suitable for

publication.

One very minor comment On page 7 line 169 you write “In other words, the observed investment probabilities allow us to derive regions with favorable conditions for a “future-proof” low-carbon cement industry in Europe.” “future-proof” seems like a bit to strong wording. Consider removing or replacing.

(Remarks on code availability)

Reviewer #2

(Remarks to the Author)
I co-reviewed this manuscript with one of the reviewers who provided the listed reports. This is part of the Nature Communications initiative to facilitate training in peer review and to provide appropriate recognition for Early Career Researchers who co-review manuscripts.

(Remarks on code availability)

Reviewer #3

(Remarks to the Author)
Since my initial comments, I have seen the following paper which proposes another policy option for a difficult-to-abate industry, such as cement; namely an industry-specific trading system. The paper suggests that these ETSs be linked internationally, but with 179 plants a cement-only trading system could be implemented by EU ETS countries. Such an ETS would provide much more specific information on emissions reduction ambition and the allowance price trajectory for the cement industry.

Haites, E. 2026. “ Using Linked Emissions Trading Systems to Transform Difficult-to-Abate Industries.” Wiley Interdisciplinary Reviews: Energy and Environment 15, no. 1: e70022. https://doi.org/10.1002/wene.70022.

(Remarks on code availability)

Open Access This Peer Review File is licensed under a Creative Commons Attribution 4.0 International License, which permits use, sharing, adaptation, distribution and reproduction in any medium or format, as long as you give appropriate credit to the original author(s) and the source, provide a link to the Creative Commons license, and indicate if changes were made.
In cases where reviewers are anonymous, credit should be given to 'Anonymous Referee' and the source.
The images or other third party material in this Peer Review File are included in the article’s Creative Commons license, unless indicated otherwise in a credit line to the material. If material is not included in the article’s Creative Commons

license and your intended use is not permitted by statutory regulation or exceeds the permitted use, you will need to obtain permission directly from the copyright holder.
To view a copy of this license, visit https://creativecommons.org/licenses/by/4.0/

Notes on resubmitting revision made to manuscript: "Decarbonizing the EU cement industry under technology and policy uncertainty"

05 June 2026

We would like to thank the reviewers for their constructive comments and suggestions that have greatly improved the quality of this manuscript. Broadly speaking, we were asked to (i) improve data transparency for our techno-economic plant and biomass assumptions, to (ii) add a sensitivity analysis to investigate the impact of plant age on our results, and (iii) to revise the discussion section to align more closely with our results. Both the manuscript and the SI have undergone a thorough revision now. We have implemented reviewers' comments throughout the whole manuscript, but specifically in the biomass, Discussion, and Methods section, where reviewers correctly pointed out that more clarity was required. We have also added two new sections to the SI: one providing more transparency with several descriptive figures on our data inputs, and one to provide a sensitivity analysis on plant age. Please see our responses to the reviewers' specific points below.

Reviewers 1 and 2

General comments

This is a concise study with an innovative methodological approach, making effective use of publicly available data. The main results highlighted by the authors are:

1. A delayed transition of the decarbonization of the cement industry occurs under conditions of low confidence and low stringency in the EU ETS.

2. Cement plants with good access to CO2 transport infrastructure (coastal or waterway proximity) are more likely to transition earlier.

3. Biomass plays a major role in the transition of the cement industry and provides potential for carbon dioxide removal (CDR).

While the first two findings largely confirm previous work (see e.g. Emanuelsson et al., 2025; Favier et al., 2018; Obrist et al., 2021; and Rootzén and Johnsson, 2011), the third finding represents a novel point of view and potentially significant contribution. However, the treatment and representation of biomass should be further strengthened to increase the credibility of the paper through clearer articulation of key assumptions and supporting evidence (see more detailed comments on this below).

Reply: We thank the reviewers for these initial comments. We agree that the discussion of the role of biomass for the cement industry has quite some potential in this study, but it is clear that some additional work was needed to strengthen our approach to this end. In the following, we hope we will convince the reviewers that we have done so both through the provision of additional data and several changes throughout the entire manuscript. We address the reviewers' specific comments in detail below.

2. Significance and relation to existing literature

The work is of clear relevance to the decarbonization of the cement sector and related industrial transitions. The methodological innovation, particularly the integration of expert elicitation with site-level techno-economic modelling, adds value to the field. However, the results largely reinforce established insights (e.g., the influence of ETS policy design and infrastructure access), and the

connection to existing literature could be better developed through more explicit referencing, discussion and comparison.

Overall, the contribution lies more in the methodological framework than in the novelty of the findings.

Reply: The reviewer is right in pointing out that the influence of the ETS and infrastructure access in decarbonising the European cement industry has been established in the literature, and we have added additional references to the manuscript to reflect this better. However, we believe our methodological contribution not only lies in the combination of expert elicitation with a bottom-up economic model, but also in the systematic consideration of *the role of uncertainty* in ETS prices, infrastructure deployment, and CAPEX developments on decarbonising investment in the European cement industry. While our results are also in line with previous work, the real-options approach allows us to go a step further in reflecting actors' *expectations on probabilities of future states* of the investment environment. None of the studies recommended by the reviewer have systematically addressed this point, and we believe it is a major contribution to the literature that needs to be highlighted. We have slightly amended our introduction to this end.

3. Support for conclusions and claims
The conclusions are generally supported by the presented results, but additional evidence and clarification would improve their robustness. In particular, assumptions around biomass availability, cost development over time, and process-specific limitations should be made more transparent. The authors appropriately reference the respective data sources from which the data were collected, but without additional information (e.g., in the supplementary material), it is difficult for the reader to understand how the data have been used and how different assumptions influence the result. We also don't find a proper description of what type of biomass you assume to be available, you mention agricultural biomass, but when looking at Figure 4 and the potential for Sweden we assume that you also include forestry biomass.

Further, the distinction between how much biomass is currently used in the industry (in the BAU scenario since all fuel today are not of fossil origin) and future use (BIO scenario) could be clarified. Specifically in relation to Figure 2a and 2b.

We thank the reviewers for this comment, which rightly points out that our biomass assumptions required more transparency. We have taken three concrete measures to deliver in this regard, and have adapted both our manuscript and the SI accordingly.

First, we now explicitly show the types of biomass we assume to be available from the ENSPRESO data set. We have included an additional Supplementary Figure 4, as well as a Supplementary Table 5 in the SI that provide more clarity on which country-level potentials across all 17 biomass types are included. In the process, we realised that we had a typo in the manuscript, and we thank the reviewers for the opportunity to also correct this minor error.

Second, we make our biomass cost assumptions more transparent now. While the biomass cost section in Methods already gives a high-level overview of our approach, we have added an additional Supplementary Figure 5 to the SI that explains how we derive our assumptions on price formation for biomass as a combination of the output from REMIND and the ENSPRESO data base.

We have adapted the corresponding part in our Methods, which now reads as follows:

"To introduce geographical differentiation, we combined these with country-level biomass cost estimates from the ENSPRESO database, which aggregates costs across 17 biomass feedstocks at the

national level. We refer the reader to Supplementary Figures 4 and 5, as well as Supplementary Table 5 for additional information on our biomass assumptions."

Lastly, regarding the distinction between current and future biomass use, we find that the cement sector can be expected to use between 742 and 848 PJ (7-8% of 10600 PJ) of biomass in 2050 (see Figure 4). Combining several data points for the sector's biomass use today[1–3], we calculate that roughly 90 PJ of biomass are currently in use: EU cement production was at roughly 160 Mt in 2023[3], which translates to 120 Mt of clinker[1]. Average biomass shares of 17-22% in thermal energy have been reported[1–3], from which we assume 0.75 GJ biomass per t of clinker. This is leaves us with 90 PJ of annual biomass use for cement production. That would mean that an eight to nine-fold increase of biomass use would be required in the European cement sector between 2025 and 2050. We have consequently included the following sentence in the section on biomass:

"Assuming a current biomass use from cement of approximately 90 PJ per year across Europe, we project a potential eight- to nine-fold increase until 2050."

Further detail on site-specific cost assumptions (OPEX, CAPEX, transport and storage costs) would strengthen credibility. We for example find that the OPEX assumptions are not well-listed in the paper, does all plants have the same OPEX or do they differ, and in such case how and why?

Reply: We are grateful to the reviewers for raising this critical point. To address it, we have created a new section with several figures in the SI that provides more transparency on OPEX, CAPEX, and transport and storage cost assumptions. At first, however, we believe it is helpful to go back to the manuscript to understand the basic structure of our plant-specific data. In the Introduction, we state how we distinguish between different plants with regard to CAPEX and OPEX:

"In the model, plant characteristics are varied across four dimensions: production capacity, CO2 transport and storage costs – which we refer to as transport costs from here on, financing costs, and local biomass costs (cf. Methods)."

The "Plant and techno-economic data" subsection in our Methods then gives a more detailed description of how we identify the 179 integrated cement plants in our set, and how we match them with (i) a CO2 transport and storage cost data set by Clean Air Task Force, (ii) country-specific biomass cost data, (iii) nameplate capacity from a geoasset data base by the Spatial Financial Initiative, which we use to represent economies of scale, as well as (iv) cost of capital via the plants' ownership structures from Orbis and Bloomberg. The former two characteristics let us distinguish plants in terms of OPEX, the latter two in terms of CAPEX. All other cost assumptions are symmetrical across all plants. To summarise our approach also in the Methods section, the following was inserted:

"Based on available data regarding plant characteristics, we distinguish between cement facilities across four dimensions: CO2 transport costs, production capacities, cost of capital, and country-specific biomass cost. All other techno-economic parameters, both in terms of cost and technology-specific operating parameters, are assumed to be homogenous across all plants."

We do acknowledge, however, that the lack of plant-specific OPEX data poses a limitation to our study, which we now flag in the Discussion section as an avenue for future research:

"Future work can contribute to more accurately identifying such competitive plants by increasing installation-specific data transparency, specifically on OPEX and plant age."

Our new section Supplementary Methods 2 in the SI now provides more transparency on the results of this data pipeline. In the section, we report both the distribution of transport and storage cost, as well as our scaling process and the resulting CAPEX distribution across all 179 plants in the set. As described above, we have also included a subsection on biomass that lays out our assumptions on potentials and cost. We hope the combination of these additions now provides sufficient clarity on our CAPEX, OPEX, and transport and storage assumptions.

We also greatly miss a discussion on the technology choices in the paper (see e.g. Cavalett which is already referenced in the paper). The authors does not acknowledge potential abatement options beyond CCS, fuel switching from fossil to biomass, and the combination of those two (BECCS). We think it is important to highlight that decarbonization needs to occur along the full value chain and that there are more options to decarbonization than just adding an end-of-pipe type of solution such as CCS (even though we agree that CCS is a highly important mitigation option in the cement industry) (see e.g. Habert et al., 2020). But there are/will be other mitigation options available such as electrification to remove the fuel emissions (which would then cancel out the whole CDR part of this paper), demand reduction through Supplementary Cementitious Materials (SCMs), demand side reduction, and so on. Again, we strongly agree that CCS is a main mitigation option for the cement industry but this exclusion of other mitigation options should be justified and proper motivated in the paper.

Reply: We thank the reviewers for pointing out this important limitation. With the plant-level approach of our ROV model, we put an emphasis on geographical patterns of investment at distinct production sites, but need to abstract from broader abatement measures across the whole value chain. The reviewers' comment on kiln electrification is also very valid, and there was a clear need to motivate our focus on CCS. We have now added the following sentences to the Introduction to reflect these points:

"Our plant-level focus allows us to evaluate geographical patterns of low-carbon cement investment, but requires us to abstract from additional abatement options in the construction value chain such as Supplementary Cementitious Materials or demand-side reduction. In addition, we exclude kiln electrification from the analysis because of its inability to address process emissions, as well as its relative technological immaturity."

4. Data analysis, interpretation, and conclusions
The analysis appears technically sound and the data treatment is carefully executed. However, some assumptions, particularly those affecting investment behaviour, require clearer justification. For instance:
• How do firms form expectations about ETS prices, CCS costs, and biomass availability?
• Do they have perfect foresight or limited information?
• How are market uncertainties (e.g., demand reduction, clinker substitution, sufficiency measures) represented?
Addressing these points would enhance the credibility of the behavioural dimension of the model. The paper could also benefit from a more explicit discussion of the model's limitations, including factors outside plant owners' control (e.g., infrastructure buildout, policy readiness, or bilateral agreements for CO2 transport).

Reply: We thank the reviewers for these comments, which broadly speak to two aspects: the behavioural dimension, and how uncertainty is represented with our approach.

For the behavioural dimension in those aspects where we have uncertainty explicitly represented (EU ETS prices, CO2 pipeline and storage site availability, CAPEX), firm's expectations are reflected through the jump probabilities between potential states of the future. This is described in the section on *Uncertain investment determinants*. We elicit those probabilities from interviews with practitioners, i.e. aim to reflect the firm's expectation through the expectation of respective decision-makers. To clarify this point, we have added the following sentence to this section:

"For the three uncertainties represented, we hence elicit firms' expectations of the future investment environment from interviews with industry practitioners."

For those three aspects where uncertainty is a systematic part of the analysis, one could indeed interpret our approach as firms having limited information, but they make their investment decision based on their expectations of possible future states of the investment environment. Over time, the uncertainty on EU ETS prices, CO2 pipeline and storage availability and CAPEX resolves, which is where the value of waiting in the ROV model stems from. We believe that the aspects mentioned in the comment's last sentence (infrastructure buildout, policy readiness, bilateral CO2 transport agreements) are therefore reflected in actors' expectations of future states of the world in terms of the EU ETS price and CO2 pipeline/storage availability.

All other investment parameters (such as plant size and cost of capital) are exogenous inputs to the model based on empirical data points from a variety of data bases (see above). Country-specific biomass availability, which we take from the EU JRC's ENSPRESO data base, is an example mentioned in the reviewer's comments that falls into this category. Such parameters could be interpreted as firms having "perfect foresight" in a sense that we do not implement any additional uncertainties for these aspects in the model.

If our specific goal were to investigate perfect foresight vs. myopic decision-making, which could be another angle through which we could envision the issue of "perfect foresight" being approached, we would have likely chosen a rolling horizon approach with a conventional discounted cash flow model. However, this would go beyond the scope of our study which puts an emphasis on the role of uncertainty in cement plants' investment decisions.

Lastly, by abstracting from abatement measures further down the value chain, we also neglect demand-side uncertainties as mentioned in the reviewer's third bullet point (demand reduction, clinker substitution, sufficiency measures). We agree that this is a limitation of our approach, which was designed to assess the investment decision at a plant level. We have therefore addressed this point and changed the manuscript accordingly (see reply above).

5. Methodology and standards
The methodology is innovative and meets high scientific standards. The integration of expert elicitation is a welcome feature and enhances the credibility of input assumptions. However, certain aspects could be elaborated to improve reproducibility and accessibility:

• Clarify cost assumptions (FOAK vs. NOAK, learning rates). You are showing how the investment cost is assumed to reduce over time in Figure 2c, but we did not find any stated assumptions about learning rates assumed to achieve those curves.

Reply: Thanks to the reviewers for the opportunity to discuss our CAPEX assumptions more in detail. Obviously, empirical evidence on CCS deployment is scarce, but we have made sure that our approach is in line with both techno-economic concepts and values reported in the literature. We have amended both the manuscript and the SI to reflect this.

Technology learning is included in our model through two channels. First, the surveyed jump probabilities of the CAPEX uncertainty reflect disruptive innovation through the possibility to jump from the high to the low CAPEX path. While our cost assumptions are exogenous and we would therefore like to be careful with the notion of FOAK vs. NOAK in our model context, the jump size we display in Figure 1c is very much in line with contingency factors to distinguish between FOAK and NOAK according to Beiron and Johnsson[4].

Second, we assume a constant CAPEX reduction of 1% per year to reflect incremental learning for each of the two cost paths. Schmidt and Malhotra make the case that learning effects are likely limited for more complex systems like CCS, and even more so for brownfield investments.[5] For this incremental learning component, we have therefore opted for a cost reduction rate that is in line with conservative estimates as provided by Beiron and Johnsson.[4] In the Methods section, we have now added the following sentence with these references to provide more backing for our assumptions:

"Final capacity estimates were used to adjust investment cost assumptions based on economies of scale, and technology learning was assumed in line with literature values to reflect incremental innovation over time (Fig. 1c)."

In addition, we do not only report our CAPEX assumptions in Fig. 1c, but have now explicitly included our reasoning for these assumptions in Supplementary Methods 2, which was added for this revision. We are confident that the combination of additions made to the manuscript and the SI now make our cost assumptions much more transparent, and we thank the reviewers again for prompting us to make this improvement.

• Specify how transport infrastructure development is treated and who makes related investment decisions. Again, you show the input you regarding the transport costs use in the modelling in Figure 2b, but the assumptions made need to be clarified. What transport modes do you allow for (road, rail, barge, pipeline)? You consistently mention "available infrastructure" but what do you actually mean by that (physically in place? That it can take more load? Or something else?)? And again, is that infrastructure "availability" expanding over time, and is such case how and who takes that decision/investment?

Reply: We agree with the reviewers that our current wording can be misleading and thank them for the opportunity to clarify. The CO2 transport cost assumptions described in Figure 1b) come from the CCS Cost Tool by the Clean Air Task Force (CATF) as referenced in the manuscript. CATF's methods state that costs depend on a plant's specific location within Europe and imply any combination of CO2 transport modes via pipeline, ship, barge, and rail. However, the two cost scenarios can be distinguished by the availability of CO_2 pipelines and a range of potential geological storage sites, which in the low-cost case are assumed to exist both on- and offshore more widely.

This had already been described in the Methods section, but we have specified the role of pipeline availability and the development of additional storage sites now not only in this part of the manuscript, but for all mentions of "infrastructure" that refer to the CATF data set.

We would like to highlight that the data set was chosen due to its ability to reflect both ends of actors' expectation on the future of CO2 transport in Europe. We were therefore able to neatly complement it with our interview approach: More pessimistic interview partners in terms of CO_2 pipeline and storage infrastructure were able to reflect their expectations in lower jump probabilities from the high-cost scenario (which is our starting point) to the low-cost scenario,

implying that pipelines would be built – and hence CO_2 transport cost reductions would materialise – rather later than sooner.

• Provide more detail on how site-specific costs are calculated and applied. In the methodology you mention “kiln fuel mixes, biomass substitution, and thermal efficiency”, are they assumed the same for all plants or different? And what are your assumptions?

Reply: We thank the reviewers for the opportunity to improve on clarity also in this regard. As already described in our reply above, we can distinguish plants across four dimensions: capacity, CO2 transport and storage costs, finance costs, and country-level biomass costs. All other exogenous parameters, such as kiln fuel mixes, potentials for biomass substitution, and thermal efficiency, are taken from the European Cement Research Academy and Cemsuisse.[6,7] Those assumptions, for each technology option respectively, are assumed to be homogenous across all plants. Our new section Supplementary Methods 2 now provides a number of figures and explanations to improve transparency on the assumptions taken.

In addition to changes on the manuscript that we have already introduced above, we have complemented the respective paragraph in Methods to also reflect this comment by the reviewers:

“Based on available data regarding plant characteristics, we distinguish between cement facilities across four dimensions: CO_2 transport costs, production capacities, cost of capital, and country-specific biomass cost. All other techno-economic parameters, both in terms of cost and technology-specific operating parameters, are assumed to be homogenous across all plants. […] Technical specifications for kiln fuel mixes, potential for biomass substitution, and thermal efficiency were assumed to be technology-specific but homogenous across plants and based on academic and gray literature.”

While the mathematical formulation is thorough, the description is rather dense in the supplementary material. Simplifying or explaining key model components in more intuitive terms would make the paper more accessible to a broader audience. Currently, the methodological details are spread out in the main section, methodology, and supplementary material, which made it quite difficult to get a proper grasp of the full methodology even after reading everything. Our recommendation is to ask a colleague of yours that is not familiar with the methodology to read the paper to give feedback on how to improve the methodological description. Again, we think that it has thorough mathematical formulations but that you could appeal to a much broader audience by explaining it in more intuitive ways. But we also want to acknowledge that you have attempted to do this through several methodological illustrations (for example Figure 2e, and in the supplementary material).

Reply: We appreciate the reviewer’s feedback on our model description, specifically in the SI. We have consequently tried to (i) introduce the method in the Introduction without going into detail, (ii) give a high-level overview of the uncertainties and the ROV in the following section, and (iii) leave the detailed description to the Methods section. Given the word count, we are not able to go more into detail in the main manuscript, and have consequently moved the mathematical formulations to the SI.

However, we have followed the reviewers’ advice and asked colleagues for feedback on the model description. While we have received positive feedback on Figures 1 and Supplementary Figure 6, we have now added a more intuitive summary to Supplementary Methods 3. It reads as follows:

"We develop a ROV model to illustrate the decarbonization investment behavior of all 179 integrated cement plants across Europe. Since we aim to systematically assess the impact of uncertainties, we expand on conventional discounted cash flow approaches by taking option values of potential investments into account. To do so, we introduce three uncertainties in the investment environment: (i) ETS prices, (ii) CO_2 pipeline and storage availability (reflected in CO_2 transport cost), and (iii) CAPEX. For each of these three uncertainties, we derive a reference path from the literature as a starting point. In a nutshell, this starting point (i.e. the investment environment today) is characterized by ambitious ETS prices, but higher cost for CO_2 transport and storage, as well as higher CAPEX. Uncertainty is then represented as follows: As time progresses, future states of these three characteristics could result in an irreversible jump from the reference path to an alternative price/cost path. From our interviews with practitioners, we construct two scenarios -- NZ2050 (i.e. more optimistic) and Delayed (i.e. rather pessimistic) -- for how respective jump probabilities develop over time. Given these uncertainties, the model allows us to quantify the option values of waiting and add-on investments, which are taken into account on top of the net present value to arrive at the investment decision."

We hope the reviewers agree that this additional description, together with the illustrations we provide through Figures both in the main manuscript and the SI, now provide sufficient clarity to communicate the method to a wider audience.

6. Reproducibility and transparency
The study provides a relatively detailed methodological description, but some critical assumptions remain implicit. Providing more explicit information on data sources, parameter ranges, and decision rules would enhance reproducibility (as previously mentioned). References to all data sources should be complete and include publication details to facilitate verification by readers.

Reply: We thank the reviewers for raising this point, which also relates to a few previous comments made. It is clear that major improvements on data transparency were needed, which is why we have taken a number of measures to do so.

First, as described above, we have added a new section to the SI, which makes our plant-level cost assumptions much more explicit now (cf. Supplementary Methods 2). More specifically, we report the distribution of financing cost vs. CO_2 transport and storage cost, our CAPEX-assumptions including economies of scale, as well as figures on biomass potentials and costs.

Second, when it comes to decision rules, section "Payback and NPV criteria" gives an explicit description of how investment decisions are taken in the model. There are no additional overarching constraints (e.g. by the regulator) that influence those individual plant-level investments.

Lastly, the compilation of our input data is made available on the GitHub repository, together with the model code. By making our input data and code open source, we encourage other researches to reproduce the study, hoping to meet highest research transparency standards with our work.

7. Recommendation
Overall, this is a high-quality and promising manuscript. The study is well executed and presents a sophisticated methodological contribution. However, given the need for clearer justification of assumptions, improved transparency of key parameters, and stronger linkage to existing literature, we recommend that the editor invite the authors to revise their manuscript before a final decision is made.

Reply: We appreciate the reviewer's positive feedback on our method and contribution to the literature. We hope that our amendments to the manuscript, as well as our additional information and descriptive figures – specifically in the areas of biomass assumptions and techno-economic (cost) transparency – now add sufficient clarity to convince the reviewers that we also meet highest standards in terms of reproducibility.

Specific comments

Please note that while we have provided a large number of comments, these are intended as suggestions for further improvement rather than requirements. The manuscript is already strong, and not all points necessarily need to be addressed in detail.

Abstract

1. In your abstract you state that 0.8 Gt of CO2 emissions would remain by 2050. I think it helps the reader if you also state the emissions before the transition, to get a grasp of actual reductions.

Reply: We thank the reviewers for pointing our attention to this detail, which helped us realize that our wording in the abstract was not precise enough. The 0.8 Gt CO2 are cumulated emissions between 2025 and 2050 in the Delayed scenario, which is why a comparison with today's emissions would not be meaningful. We could mention the counterfactual under business-as-usual emissions here (2.1 Gt CO2 emissions in total between 2025 and 2050), which we also show later in the paper. However, we believe that the comparison to the NZ2050 scenario is actually more meaningful, because it shows the stark discrepancy between the cement sector's roles as CO2 source or sink in the overall climate policy architecture. We have hence chosen a more accurate wording for the abstract, which now reads as follows:

"[…] High confidence in favorable investment determinants could lead to widespread deployment of carbon capture and storage and biomass use, turning the cement industry into a carbon sink with 0.6 Gt CO_2 removed between 2025 and 2050 in total. Low confidence, however, could lead many cement producers to delay action, resulting in cumulated emissions of 0.8 Gt CO_2 over the same time frame. […]"

Methodology

2. The Finnsementti plant in Lappeenranta seems to be missing in the data? It could be categorized according to the old activity codes in the ETS data (activity code 6 I believe).

Reply: We thank the reviewers for highlighting that the Lappeenranta cement plant is not part of the data set. As rightly pointed out by the reviewers, this specific plant reports under a different EU ETS activity code, which covers both cement clinker and lime production. Including this activity code would risk incorporating lime producers that are not cement plants, undermining the reliability of the selection algorithm as described in the "Plant techno-economic data" subsection of our Methods. We have adapted the Methods section to make this choice explicit:

"While some individual cement plants report under different activity codes and are therefore not part of the data set (e.g. the Finnsementti plant in Lappeenranta, Finland), this approach provides us with a systematic and reproducible selection algorithm that avoids contaminating the sample with non-cement producers."

We hope the reviewers agree with the choice of prioritising a robust selection algorithm over having a more "complete" data set at the risk of being contaminated with plants outside the scope of our paper.

Main

3. Statement: Line 23 “Combining CCS with a fuel switch to biogenic energy (BECCS) could permanently remove CO_2 from the atmosphere.”. Comment: even though you say “could”, it would be beneficial to state here under what circumstances this applies.

Reply: We agree with the reviewers that a political discussion needs to be held regarding the degree to which certified biomass will continue to receive a zero-rating in an ETS context. However, the combination of bioenergy with carbon capture and permanent storage (which is implied by CCS) results in a net removal of atmospheric carbon dioxide. We have adapted the sentence to reflect this accordingly.

4. Statement: Line 24-25 “Therefore, cement production could become an important contributor to carbon dioxide removal (CDR)”. Comment: it would help the reader if you could also state some potential here for how much CDR the cement industry could actually contribute with in relation to what’s needed. Then the reader could get an insight into whether the cement industry is an important player here or not.

Reply: Thanks for raising this very valid point, which we have implemented in the results section now. We have added the following sentence:

“In our NZ2050 scenario, the cement industry transitions to a sink in the early 2030s already and contributes 57.1 Mt of annual CDR by 2050. This means the cement industry alone could deliver half of the EU’s annual industrial CDR in 2050 as projected by the EU Commission’s 2040 target Impact Assessment.”

5. Statement: Line 28-29 “Until then, the allowance cap will continue to gradually decline every year, making EU carbon pricing increasingly onerous.”. Comment: “onerous” from an industrial actors point of view?

Reply: Yes, indeed – we have specified “for emitting installations” in the manuscript.

6. Statement: Line 31 “and over €100 billion in financing”. Comment: and how much is that in comparison to what is needed in the cement industry?

Reply: While we see why a comparison could be useful, we also believe that any estimate would be subject to large uncertainties as it would hinge on the set of assumptions used to derive it. In addition, the number of €100 billion is structurally different to scientific estimates for investment needs in the cement industry because (i) it is purely political in a sense that it was presented in communications by the EU Commission only, and (ii) it was announced for the Clean Industrial Deal as a whole. In an attempt to avoid comparing apples and oranges, we would therefore prefer to omit any comparison here. We hope the reviewers agree with our reasoning.

7. Statement: Line 33: “For the cement industry, uncertainty is particularly high:”. Comment: compared to what? Many industries are facing similar issues.

Reply: We agree with the reviewers that irreversible, bulky investments can also characterise other industries, and that those might hence face similar challenges. We have deleted the sentence from the manuscript.

8. Statement: Line 42: “First, given the landscape of cement plants today, it remains unclear to what extent the sector overall can contribute to mitigating climate change, both through emissions reductions and the potential to deliver CDR.”. Comment: but how much they can reduce their emissions isn’t really unclear? Or do you mean cumulative emissions?

Reply: While we think we understand where the reviewers are coming from with this comment, the main goal of this study is to explicitly represent uncertainties in cement plants' low-carbon transition. Against this background, we would kindly disagree with the comment made: Such uncertainties influence firms' investment decisions, and it is therefore – both from a cost-effectiveness and an efficiency perspective – not straightforward to determine how large the cement sector's contribution to fighting climate change will (or should) be. We are convinced this holds for both abatement and removal, and hope the reviewers agree with our reasoning on this point.

9. Statement: Line 50 "Second, more clarity is needed on the distributional implications of a "new economic geography of decarbonization" in the European cement industry.". Comment: what do you mean by new economic geography of decarbonization? Perhaps good to define it?

Reply: The term "economic geography of decarbonization" was coined by Zachmann and McWilliams[8], which we also cite at this position in the manuscript. We have inserted "the spatial redistribution of low-carbon production capacities across regions" to provide further clarity.

10. Statement: Line 56 "there is limited understanding of how accounting for decision-making under uncertainty influences the geographical aspects of the cement industry's decarbonization". Comment: with "geographical aspects" do you mean how the location of the cement plants would be impacted? Or could you define what you mean here since you don't discuss relocation in the paper.

Reply: We thank the reviewers for this important clarification question. As defined above now, we do indeed refer to the location of future cement plants. The reviewers are also right in pointing out that we are not able to depict actual relocation with our ROV-approach. However, it is possible to conclude from our results that regions with a higher probability of low-carbon investment offer favourable conditions for a future-proof EU cement industry. We have specified this now further at the beginning of the "Investment by country and plant" section.

"From a geographical perspective, our results highlight the risk of smaller and landlocked plants being left behind due to a combination of modelled uncertainties and adverse locational factors in a world with ambitious carbon management. In other words, the observed investment probabilities allow us to derive regions with favorable conditions for a future-proof low-carbon cement industry in Europe."

11. You often say European cement industry but you are really looking at EU plus Switzerland.

Reply: Our data pipeline described in Methods lets us include all integrated cement plants that are regulated under the EU and the Swiss ETS, unless they report under a different activity code (see our reply to point 2. above). This also includes countries which are part of the EEA such as Norway. We very much agree with the reviewers that there was a need to specify this in the manuscript, which we now do in the Introduction:

"We do so by developing a pure jump real options valuation (ROV) model to assess the decarbonization investment behavior of 179 integrated cement plants across Europe, more specifically across all countries that are regulated under the EU and the Swiss ETS."

12. By excluding non-integrated plants, how much of the total industry emissions are you neglecting?

Reply: We thank the reviewers for this important question. Non-integrated plants cover downstream steps after clinker production, i.e. are mostly covering grinding and blending. For these steps, emissions caused stem from electricity consumption (i.e. Scope 2) of the respective facilities.

The EU Commission's Joint Research Center estimates that each tonne of grey and white cement requires roughly 114 kWh of electricity .[1] At the EU's average CO_2 intensity of electricity generation in 2024 of 187 g CO2/kWh[9], this would result in grinding and blending emissions of approximately 21 kgCO_2 per kg cement. Comparing this to primary emissions of 600-1000 kg CO_2/kg cement, the non-integrated share would account for maximum 3.5% of overall emissions (however, a small mark-up for clinker transport could still come on top).

While this is a rather small share already, under the EU ETS it would be accounted for by allowances surrendered from the power sector, which is outside the scope of this work. In addition, the purpose of our study is to investigate deep-decarbonising investments, as opposed to efficiency improvements in grinding and blending. We therefore hope the reviewers agree with the assumption that emissions from non-integrated plants are negligible for the purpose of our study.

13. Statement: Line 68 "This approach enables us to estimate the value of waiting, and to identify the effect of key uncertainties on timelines for decarbonization investments in the EU cement industry, including geographical patterns and variations in producers' expectations about the future development of key investment determinants.". Comment: what do you mean with "the value of waiting"? And I am not sure what you mean with "geographical patterns" here. Please clarify.

Reply: Thanks to the reviewers for the opportunity to clarify these two points. The value of waiting is a well-established concept in the real options literature, referring to the economic benefit an investor gets from deferring an irreversible investment decision until more information about future conditions is available.[10] In our context, this means it may be rational to delay a cement plant's decarbonization investment, even if its expected net present value is currently positive. The value of waiting quantifies this benefit: it represents the additional value generated by preserving flexibility and investing only once the investment environment has become sufficiently clear – for instance, once CO_2 pipelines are in place. Our ROV model explicitly calculates this value for each plant and time step, allowing us to identify when and under what conditions a plant chooses the option to wait over immediate investment. We have specified "the value of waiting with an irreversible investment" in the manuscript now to emphasise this point.

We have addressed the question on "geographical patterns" in our replies above, and hope that our changes to the manuscript now provide sufficient clarity on this matter.

Investment patterns and emissions trajectories
14. Statement: Line 102 "In this "best-case" scenario". Comment: best from what perspective? Better to call it "low-emission scenario" or other?

Reply: We thank the reviewers for this important comment. There is certainly a need to specify, but in our view "low-emission" scenario does not describe the NZ2050 accurately either. This is because it refers to the outcome rather than the investment environment (sentiment) that drives low-carbon projects. From our interviews, it became clear that actors can have a more optimistic or a more pessimistic view of the future low-carbon investment landscape for cement. We then decided to introduce the two scenarios to reflect this distinction.

Therefore, here "best-case" was referring to a situation in which actors have more confidence – mostly in climate policy (high carbon prices) and CO_2 pipeline deployment – and therefore opt for

low-carbon investment earlier. We have rephrased it as "more optimistic" now, and hope reviewers agree that it is a more accurate framing to describe the sentiment among cement investors.

15. Swap places of Figure 2c and 2d since you explain 2d first in the text?

Reply: Thanks for this comment, which made us realise there was a missing reference to Figure 2c in the sentence before pointing to Figure 2d. We have included this reference, and the respective orders of text and subfigures are coherent now.

16. Is Figure 2b only transport cost or does it also include the cost for storage? Or have you stated that cost assumption elsewhere?

Reply: We already discuss a related comment on transport and storage costs in our reply to an earlier remark by the reviewers, but very much agree that there was a need to specify this in the manuscript. We have changed the second-to-last paragraph in the introduction to the following:

"In the model, plant characteristics are varied across four dimensions: production capacity, CO2 transport and storage costs – which we refer to as transport costs from here on, financing costs, and local biomass costs (cf. Methods)."

Our Methods section then provides additional level of detail:

"To capture site-specific cost heterogeneity, we manually matched ETS installation names with facility names in a dataset provided by the non-governmental organization CATF in May 2023, which includes georeferenced data on CO_2 transport and storage costs across Europe. […] CO_2 transport and storage costs are plant-specific and reflect estimates from the CATF data, under the "short-term infra-structure" scenario in which only limited onshore and offshore storage capacity is assumed to be available."

We hope our addition in the main manuscript now clarifies that storage costs are always included, even if we opt to write "transport costs" for brevity throughout the manuscript.

17. Figure 1c shows the specific CCS investment cost and the y-axis states that the unit is in M€ while the caption says M€ per ton of CO2 captured per hour. It could also be beneficial to the manuscript to mention this in absolute numbers (for example X M€ for a cement plant with a capacity of 1 Mt (or similar)), so that the reader doesn't have to go to supplementary materials to check the order of magnitude.

Reply: Thanks to the reviewers for noticing, we have corrected the y-axis label to $M€\ t^{-1}$ now to make it consistent with the caption. We have opted to leave an absolute investment example out of the main manuscript since the amount would quite considerably depend on the actual plant size. We hope the reviewers agree with this decision.

Investment by country and plant

18. In the caption of Figure 3 you state: "BIO investments without CCS overall make up less than 1% (cf. Fig. 2) and are therefore not shown.". But in Figure 2 BIO is always zero, is it rounded to zero?

Reply: Many thanks for pointing this out. For clarity, we have removed those 0%-labels where the value was actually zero, and now only display those where the value was indeed rounded. Please note that Panels a and b display the sum of probability-weighted nameplate capacities, which explains the observed small percentage values. The adapted caption of Fig. 2 should now clearly convey this:

“The indicated clinker production capacities are the sum of probability-weighted nameplate capacities for all 216 conceivable developments of the investment environment. Displayed 0%-labels are rounded.”

Biomass demand for cement decarbonization

19. Statement: Line 193 “Cement decarbonization may use a substantial portion of Europe’s locally available biomass, depending on the country”. Comment: I think you need to add a discussion if this is actually an efficient use of biomass resources or if it would be more beneficial (from a climate perspective) to use these green carbon atoms in other sectors. Since this is a key part of the results this needs to be put into context and problematized more clearly. e.g. what is the cement industries “willingness to pay” for biomass compared other sectors in a carbon constrained future. What does the literature say about other uses of biomass and “green” C atoms?

Reply: We thank the reviewers for raising this important issue, for which we share the reviewers’ concerns. We already touch upon this point in the sentence right after:

“BECCS could enable a net-zero and net-negative future for the cement industry and unlock the carbon (removal) value of biomass, which is considered higher than its energetic value from a societal perspective.”

At this point in the manuscript, we refer to Millinger et al., which provide system-level evidence to support the view that the specific sector in which biomass is deployed matters less than whether the carbon is captured.[11] The authors find, using a sector-coupled European energy system model, that it is not crucial in which sector biomass is used, as long as it is combined with carbon capture to enable negative emissions and feedstock for e-fuel production. This finding directly addresses the reviewers’ concern: BECCS from cement fits precisely within the category of high-value biomass applications identified by the study.

We nevertheless agree with the reviewers’ concerns in a sense that energy system models can only do so much to represent competition for biomass. We raise this issue in the same paragraph:

“However, switching large parts of cement production capacity to BECCS will put pressure on European biomass supply, warranting a comparison of future biomass demand and potential.”

This already sets the tone for the remainder of the section, in which we have decided to develop Figure 4 for exactly this reason: to assess the extent by which biomass demand from cement can be expected to impact national biosystems in the countries considered. For this estimate, we do not rely on model assumptions, but use potentials from the ENSPRESO data base (see above). We also flag this issue in the discussion section, where we do not only make the problem explicit, but propose a potential way forward:

“This demand would inevitably lead to rivalry with other sectors (e.g., heating or sustainable aviation fuels). The “simplest” economic solution to handle tradeoffs between CDR, agriculture, and ecosystem services would be to price biomass externalities appropriately, ensuring it is used where it has the highest societal value. While such a policy could change the picture we have drawn, further analysis is required, which is beyond this work’s scope.”

We therefore hope we were able to show that we do not treat biomass lightly, and that we have already attempted to make this clear in the manuscript.

20. Statement: Line 208 “While the cement industry will inevitably compete for this biomass with other sectors, cement producers have the advantage of being able to handle different types of (low-

quality) biomass". Comment: will they actually be able to compete for this biomass though? As you state in your introduction: "Cement production is particularly affected by increasing ETS allowance prices caused by the cap reduction, due to cement's high emissions intensity relative to its low general cost", the cement industry may not have much purchasing power to compete with other industries for this biomass. (See also comment above)

Reply: We thank the reviewers for this comment, which already makes the important connection to ETS prices. We would argue that cement producers' "willingness to pay" depends on ETS prices, hence the question of biomass investment is rather one of opportunity cost than one of available cash flow. Under the ETS, each producer has one of two options: to abate, or to buy allowances. Therefore, the decision to procure biomass needs to be weighed against the counterfactual of increased emissions, and therefore higher cost of compliance from allowance procurement. With the opportunity for CDR, an additional revenue stream for certificates will open up. In this case, biomass will become part of the value chain for CDR, which, given the high carbon value of biomass, is economically desirable.[11]

Discussion

21. Statement: "Second, clearer political guidance is needed on the role of cement in delivering negative emissions". Comment: but I still think it's very unclear (from a climate perspective) what role the cement industry should take in a negative emissions perspective since we don't know yet where the biomass will be of "best use" from a climate perspective.

Reply: We thank the reviewer for raising this important point. We agree that identifying the single "best use" of biomass across all sectors from a climate perspective remains a complex question. However, we would argue that, on top of the findings by Millinger et al. described above[11], two more considerations provide reasonable confidence that biomass use in cement BECCS is put to good societal use.

First, we assume that "best use" means achieving a unit of mitigation at the lowest societal cost. Under this interpretation, the EU ETS provides a powerful and well-established allocation mechanism. Currently, more than 200 Mt of emissions under the EU ETS are exempt from the surrender of allowances because they originate from certified biomass combustion.[12] As soon as biomass enters a compliance carbon market, cost-effectiveness is structurally ensured: a producer will only switch to biomass if doing so is cheaper than procuring and surrendering an ETS allowance. The volumes of biomass demand we observe in our results – even under the NZ2050 scenario – represent only a fraction of the biomass already circulating within the ETS, suggesting that the market mechanism would allocate this biomass to cement BECCS only where it genuinely represents the least-cost mitigation option.

Second, cement producers have a practical advantage in this competition: as mentioned in the manuscript, unlike many other sectors, they can utilize a wide range of low-quality biomass feedstocks that are less suitable for higher-value applications such as aviation fuels or chemicals, reducing direct rivalry with those uses.

Taken together, these arguments suggest that biomass in cement BECCS is not deployed arbitrarily, but rather in a cost-effective manner governed by market signals – precisely the kind of allocation mechanism that minimizes societal cost. While we have kept the discussion section to discuss the challenges of widespread biomass deployment, we have revised the biomass section of the manuscript to make our reasoning more explicit.

Suggested references

Emanuelsson, A. H., Rootzén, J., & Johnsson, F. (2025). Deployment of carbon capture and storage in the cement industry – Is the European Union up to shape? International Journal of Greenhouse Gas Control, 146, 104442. https://doi.org/10.1016/j.ijggc.2025.104442
Favier, A., De Wolf, C., Scrivener, K., & Habert, G. (2018). A sustainable future for the European Cement and Concrete Industry: Technology assessment for full decarbonisation of the industry by 2050. ETH Zurich. https://doi.org/10.3929/ethz-b-000301843
Habert, G., Miller, S. A., John, V. M., Provis, J. L., Favier, A., Horvath, A., & Scrivener, K. L. (2020). Environmental impacts and decarbonization strategies in the cement and concrete industries. Nature Reviews Earth & Environment, 1(11), 559–573. https://doi.org/10.1038/s43017-020-0093-3
Obrist, M. D., Hischier, R., & Horvath, A. (2021). Decarbonisation pathways of the Swiss cement industry to 2050: Long-term energy consumption and CO_2 emission scenarios. Journal of Cleaner Production, 298, 126746. https://doi.org/10.1016/j.jclepro.2021.126746
Rootzén, J., Kjärstad, J., & Johnsson, F. (2011). Prospects for CO2 capture in European industry. Management of Environmental Quality: An International Journal, 22(1), 18–32. https://doi.org/10.1108/14777831111098453

Reply: We thank the reviewers for suggesting this list of references, which we have now included in the manuscript.

Reviewer 3

The paper models the effects of uncertainties related to ETS allowance prices, carbon-capture investment costs, and the availability of CO2 transport infrastructure affect decisions by cement plants across Europe to deploy carbon capture and storage and biomass use to reduce emissions and potentially turn the cement industry into a carbon sink.

I have two substantial comments.

The first relates to the impact of not considering the age of the plants and kilns. This appears to be due to a lack of plant-specific data. It seems likely, however, that a plant whose kiln needs to be replaced within 5 years would attach different weights to the uncertainties than a plant whose kiln has a projected lifespan of 20 years. If age/remaining lifespan data is not available, I suggest sensitivity analyses for a representative plant(s) assuming different ages to assess the impact on investment decisions.

I suspect that the effect would be accelerated adoption of CCS for plants with a remaining life of a decade or less and slower adoption for newer plants. Given the high rate of adoption of CCS as the initial mitigation option especially for the NZ2050 scenario, the results may be very similar even after adjusting for the age of the plant/kiln. Regardless I believe plant/kiln age should be explicitly addressed in some way.

Reply: Many thanks to the reviewer for raising this point on plant age, which prompted a valuable addition to our analysis. We went back to our original dataset to check again if a detailed analysis would be possible, and found that indeed we do not have plant-specific data on the start of production available as it is not reported for the majority of plants.

We were nonetheless keen to test the reviewer's hypotheses, so we decided to approximate plant age through our CAPEX assumptions: Since the investment decision is taken relative to re-investment in a conventional facility, the effective (relative) CAPEX of older plants would be lower, and that of newer plants higher, respectively. While one could in principle adjust uncertainty weights to reflect plant age as suggested by the reviewer, we believe the CAPEX channel has the advantage of a clearer economic interpretation and a more direct link to observed investment levels. We consequently used this mechanism and created a new section in the SI, where we vary our baseline CAPEX assumption by 25% upwards and downwards. We are aware that this is a stylized assumption, but we expected it to be sufficient to demonstrate the direction of the effect.

Indeed, the findings from the sensitivity analysis fully support the reviewer's intuition: Our results for the NZ2050 scenario are very robust both for older and newer plant fleets, indicating that high confidence in the investment environment provides plant operators with a business case that dominates the age sensitivity. In this case, plant age does not play a role.

In the Delayed scenario, however, we show that an initially older fleet of plants would both accelerate the transition slightly and end up at a lower emission level. For an initially newer plant fleet, the effect goes in the opposite direction (as expected by the reviewer): Plants invest slightly later, and the share of plants that do not invest at all in the Delayed scenario increases from 17% to 26% in comparison to our baseline CAPEX assumptions.

The results consequently confirm the reviewer's intuition on the direction of effects while showing that plant age is unlikely to change the key conclusions of the paper, particularly under the NZ2050 scenario. We have added a respective paragraph in the Results section:

"We lack installation-specific data on plant age, but conduct a stylized sensitivity analysis in Supplementary Note 1. The NZ2050 scenario results are robust both for older and newer plant fleets, indicating that high confidence in the investment environment provides plant operators with a compelling business case regardless of plant age. In the Delayed scenario, however, an initially older plant fleet both accelerates the transition slightly and lands at a lower overall emission level; a newer plant fleet shows the opposite effect."

The second relates to the emphasis on the ETS in the short section on policy implications. The paper asserts that "the EU needs to uphold and strengthen the credibility of its carbon market, … reach consensus … on a clear target picture for the time after an "ETS endgame, [and] … support the ETS credibly through the implementation of the Carbon Border Adjustment Mechanism (CBAM)."

Cement industry emissions account for less than 10% of the emissions covered by the ETS. An ETS provides an incentive for all covered sources to implement reductions whose marginal cost is less than the allowance price. Emission reductions through cement plant CCS are likely to be costly relative to the mitigation options available to other ETS participants. Thus relying on the ETS price to drive cement industry CCS adoption is likely to have limited impact until the lower cost options available to other participants are exhausted.

To me the message from the modelling results is clear – CCS is the first step toward cement industry emissions reductions and implementation of this technology can begin immediately. The policy implications, then, are equally clear; policies that stimulate early adoption of CCS by cement plants. The paper mentions two such policies -- Carbon Contracts for Difference (CCfDs) and a coherent long-term strategy for CO2 infrastructure – but buries them in the middle of the ETS discussion. Additional policies could include regulations that require new cement plants to install CCS and, probably with a lag, policies to increase the supply of biomass.

Revise the policy discussion to draw on the modelling results.

Reply: We thank the reviewer for this important comment, which we largely agree with, and which has led us to substantially revise the policy discussion.

The reviewer is correct that the ETS price alone is unlikely to drive cement CCS adoption in the near term, since other covered sectors retain cheaper abatement options that will be exhausted first. We have restructured the policy discussion to lead with the instruments most relevant to near-term action, specifically Carbon Contracts for Difference (CCfDs) and a coherent long-term CO_2 infrastructure strategy – which our results identify as one of the two most influential investment determinants. As suggested by the reviewer, we now also explicitly include regulatory requirements for CCS installation at new cement plants, as well as biomass supply policies as a necessary complement to BECCS deployment in the medium term.

At the same time, we believe that ETS credibility remains an essential part of the policy discussion, and we respectfully ask the reviewer to consider retaining this emphasis. Our results provide direct support for our ETS-related statements: the contrast between the NZ2050 and Delayed scenarios shows that producer confidence in a sustained high carbon price is one of the strongest drivers of the timing and scale of low-carbon investment. This result is also confirmed by Emanuelsson et al.[13], who show that emissions reductions in the cement sector can only stay in line with the EU's climate targets if EU ETS prices are sufficiently high. The reviewer rightly points out that the ETS is not the trigger for early action, but it remains the backbone of the long-run investment case. Failing to credibly signal its trajectory beyond 2040 would undermine even the near-term policies listed above,

since CCfDs and infrastructure investments ultimately depend on expectations about future carbon prices. We have revised the text to make this sequencing explicit: near-term support instruments first, with ETS credibility framed as the necessary long-run complement as opposed to the primary near-term driver.

References

1. Marmier, A. *Decarbonisation Options for the Cement Industry*. https://publicationstest.jrc.cec.eu.int/repository/handle/JRC131246 (2023) doi:10.2760/174037.
2. Cavalett, O., Watanabe, M. D. B., Voldsund, M., Roussanaly, S. & Cherubini, F. Paving the way for sustainable decarbonization of the European cement industry. *Nature Sustainability 2024 7:5* **7**, 568–580 (2024).
3. Cement Europe. *Cembureau Key Facts & Figures*. (2025).
4. Beiron, J. & Johnsson, F. Progressing from first-of-a-kind to Nth-of-a-kind: Applying learning rates to carbon capture deployment in Sweden. *International Journal of Greenhouse Gas Control* **137**, 104226 (2024).
5. Malhotra, A. & Schmidt, T. S. Accelerating Low-Carbon Innovation. *Joule* **4**, 2259–2267 (2020).
6. European Cement Research Academy. *The ECRA Technology Papers 2022 - State of the Art Cement Manufacturing - Current Technologies and Their Future Development*. https://ecra-online.org/research/technology-papers (2022).
7. Cemsuisse. *Kennzahlen*. (2021).
8. Zachmann, G. & McWilliams, B. A new economic geography of decarbonisation? Preprint at https://www.bruegel.org/blog-post/new-economic-geography-decarbonisation (2021).
9. European Environment Agency. Greenhouse gas emission intensity of electricity generation in Europe. *Indicators* (2025).
10. Dixit, A. K. *Investment under Uncertainty*. (Princeton University Press, Princeton (N.J.), 1994).
11. Millinger, M. *et al.* Diversity of biomass usage pathways to achieve emissions targets in the European energy system. *Nature Energy 2025 10:2* **10**, 226–242 (2025).
12. European Commission. *Report from the Commission to the European Parliament and the Council on the Functioning of the European Carbon Market in 2023*. (2024).
13. Emanuelsson, A. H., Rootzén, J. & Johnsson, F. Deployment of carbon capture and storage in the cement industry – Is the European Union up to shape? *International Journal of Greenhouse Gas Control* **146**, 104442 (2025).

Notes on second re-submission of manuscript: “Decarbonizing the EU cement industry under technology and policy uncertainty”

20 July 2026

We would like to thank the reviewers for their constructive comments during the previous review round, which greatly improved our manuscript. We are delighted to read that all reviewers are content with the respective changes, and have gladly addressed the remaining comments.

Reviewers 1 and 2

Thank you for the opportunity to review the revised manuscript. We believe the authors have addressed the comments and questions thoroughly and have improved the manuscript accordingly. In our view, the manuscript is now suitable for publication.

One very minor comment On page 7 line 169 you write “In other words, the observed investment probabilities allow us to derive regions with favorable conditions for a “future-proof” low-carbon cement industry in Europe.” “future-proof” seems like a bit to strong wording. Consider removing or replacing.

Reply: We thank reviewers 1 and 2 for the positive feedback, as well as the remaining comment on our wording in the “Investment by country and plant” subsection of our Results. We agree with the reviewers and have consequently deleted “future-proof” from this sentence.

Reviewer 3

Since my initial comments, I have seen the following paper which proposes another policy option for a difficult-to-abate industry, such as cement; namely an industry-specific trading system. The paper suggests that these ETSs be linked internationally, but with 179 plants a cement-only trading system could be implemented by EU ETS countries. Such an ETS would provide much more specific information on emissions reduction ambition and the allowance price trajectory for the cement industry.

Haites, E. 2026. “ Using Linked Emissions Trading Systems to Transform Difficult-to-Abate Industries.” Wiley Interdisciplinary Reviews: Energy and Environment 15, no. 1: e70022. https://doi.org/10.1002/wene.70022

Reply: We thank the reviewer for this reference with an additional policy proposal, which we now include in our Discussion section with the following sentence.

“To safeguard the integrity of the ETS, the EU should reach consensus on a clear target picture for the time after an “ETS endgame.” One recently proposed alternative foresees the international linking of industry-specific trading systems, which could provide a more targeted approach for specific sectors such as cement. Additionally, the EU needs to uphold a functioning regime against carbon leakage, more specifically, to support the ETS credibly through the implementation of the Carbon Border Adjustment Mechanism (CBAM).”
